# The SARS-CoV-2 Programmed −1 Ribosomal Frameshifting Element Crystal Structure Solved to 2.09 Å Using Chaperone-Assisted RNA Crystallography

**DOI:** 10.1021/acschembio.1c00324

**Published:** 2021-07-30

**Authors:** Christina Roman, Anna Lewicka, Deepak Koirala, Nan-Sheng Li, Joseph A. Piccirilli

**Affiliations:** † Department of Biochemistry and Molecular Biology, The University of Chicago, Chicago, Illinois 60637, United States; ‡ Department of Chemistry, The University of Chicago, Chicago, Illinois 60637, United States; § Department of Chemistry and Biochemistry, University of Maryland Baltimore County (UMBC), Baltimore, Maryland 21250, United States

## Abstract

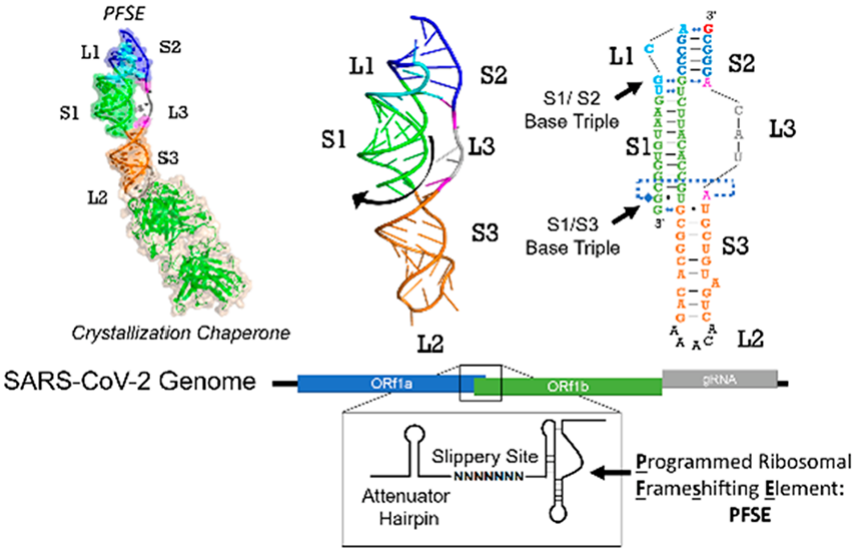

The programmed −1 ribosomal frameshifting element (PFSE) of SARS-CoV-2 is a well conserved structured RNA found in all coronaviruses’ genomes. By adopting a pseudoknot structure in the presence of the ribosome, the PFSE promotes a ribosomal frameshifting event near the stop codon of the first open reading frame Orf1a during translation of the polyprotein pp1a. Frameshifting results in continuation of pp1a via a new open reading frame, Orf1b, that produces the longer pp1ab polyprotein. Polyproteins pp1a and pp1ab produce nonstructural proteins NSPs 1–10 and NSPs 1–16, respectively, which contribute vital functions during the viral life cycle and must be present in the proper stoichiometry. Both drugs and sequence alterations that affect the stability of the −1 programmed ribosomal frameshifting element disrupt the stoichiometry of the NSPs produced, which compromise viral replication. For this reason, the −1 programmed frameshifting element is considered a promising drug target. Using chaperone assisted RNA crystallography, we successfully crystallized and solved the three-dimensional structure of the PFSE. We observe a three-stem H-type pseudoknot structure with the three stems stacked in a vertical orientation stabilized by two triple base pairs at the stem 1/stem 2 and stem 1/stem 3 junctions. This structure provides a new conformation of PFSE distinct from the bent conformations inferred from midresolution cryo-EM models and provides a high-resolution framework for mechanistic investigations and structure-based drug design.

## Introduction

The historic and deadly COVID-19 pandemic is caused by the Severe Acute Respiratory Syndrome Coronavirus 2 (SARS-CoV-2). Researchers around the world are searching for treatments for this catastrophic disease, in part by targeting drug design efforts toward the structured RNA elements in the nearly 30 kb RNA genome of the virus. However, a lack of high-resolution, three-dimensional structural information about structured regions of the genome make development of drugs to target them difficult. Computational modeling and structural probing techniques are able to identify structured regions within RNAs and can suggest whether an RNA element might contain a pocket sufficient for ligand binding, but these estimations often lack certainty about the chemical arrangement of binding pockets.^1,2^ By contrast, broad screens of RNA binding chemicals do not require high resolution structural information as a starting point and can yield lead molecules, but these chemicals are rarely drug-like due to their toxicity, lack of cell permeability, or lack of bioavailability.^1^ Experimentally derived structures of viral RNA elements can provide another route to drug discovery. Existing drugs can be screened against experimentally determined structural models using structural dynamics simulations to identify potential binders.^3^

One potentially druggable RNA target in the SARS-CoV-2 genome is the programmed −1 ribosomal frameshifting element (PFSE). Thus far, it has been shown to bind the ligand 2-{[4-(2-methyl-thiazol-4ylmethyl)-[1,4]diazepane-1-carbonyl]-amino}-benzoic acid ethyl ester (MTDB), and in cell culture this ligand can compromise ribosomal frameshifting and inhibit viral replication by three orders of magnitude.^3−8^ Chemical probing and homology modeling consistently predict this programmed frameshifting element to form a stable and well-ordered three-stemmed H-type pseudoknot.^8−14^ For structural biologists, its small size also makes it a promising candidate for X-ray crystallography. Thus, we saw an opportunity to support the drug design process and mechanistic investigations by generating a high-resolution structure of the programmed frameshifting element using our lab’s method of chaperone-assisted RNA crystallography.

**Figure 1:**
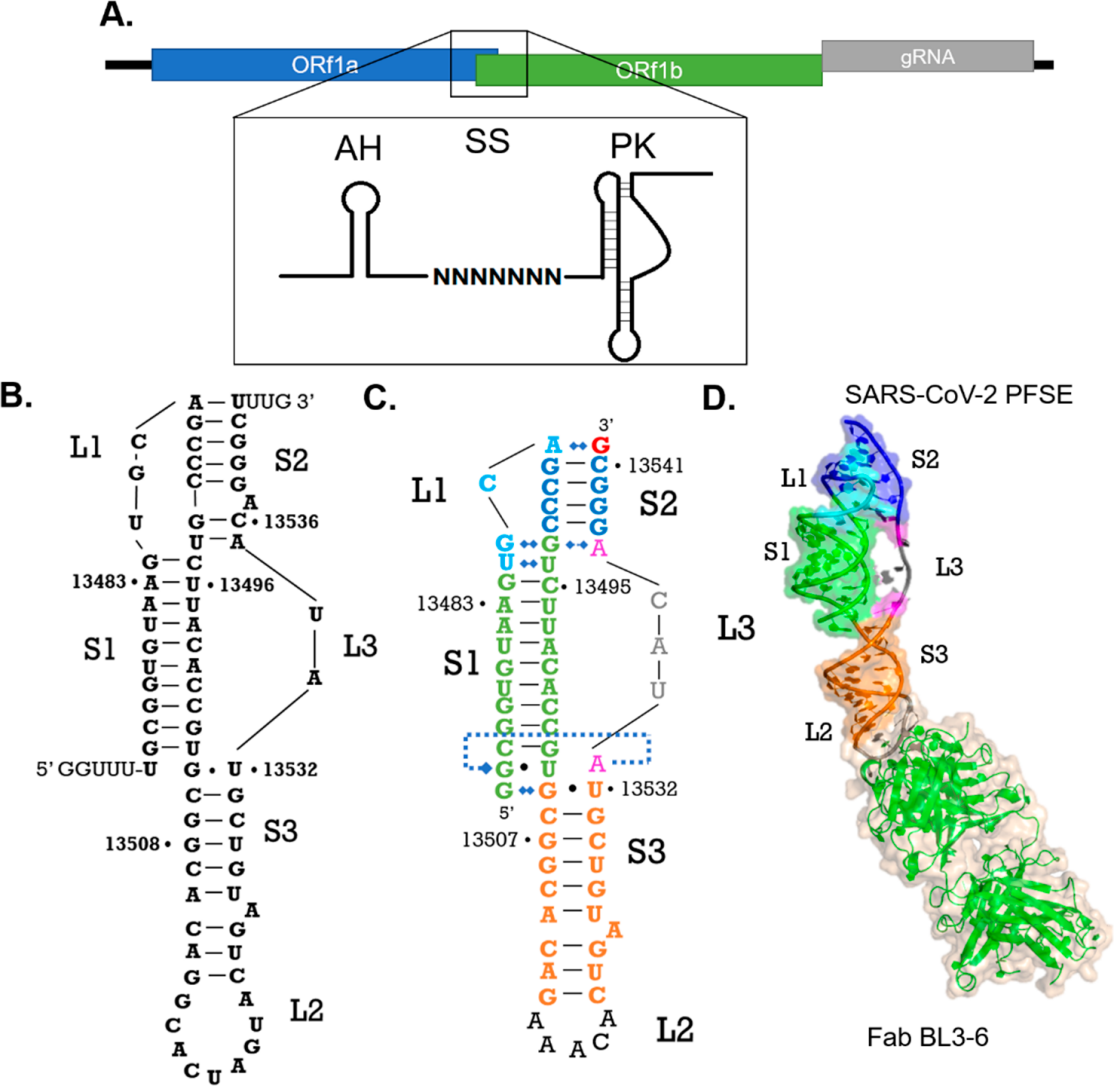
Overall structure of SARS-CoV-2 programmed −1 ribosomal frameshifting element pseudoknot. (A) Diagram of frameshifting element relative to SARS-CoV-2 genome. AH indicates the attenuator hairpin, SS indicates the slippery site and PK indicates the pseudoknot structure.^6^ (B) Predicted secondary structure of the programmed frameshifting element.^6^ (C) Secondary structure derived from the crystal structure; Stem 1 colored green, Loop 1 colored cyan, Stem 2 colored navy, Loop 3 colored magenta with nucleotides lacking density colored gray, Stem 3 colored orange, Loop 2 mutant pentaloop colored black, nucleotide 13542 shown in red was added via nonspecific addition in in vitro transcription reaction. (D) Crystal structure of the SARS-CoV-2 frameshifting element bound to Fab BL3–6 through its mutated loop structure shown as cartoon and transparent surface; surface excluded for nucleotides with no electron density in Loop 3. Color scheme of crystal structure matches that of the secondary structure in (C).

Ribosomal frameshifting from Orf1a to Orf1b is a critical step in coronavirus propagation.^15,18^ Orf1a and its out-of-frame continuation Orf1b are the first open reading frames to be translated directly from the SARS-CoV-2 RNA genome upon infection and disassembly (Figure 1A). They encode the nonstructural proteins (NSPs) 1–16, which are involved in evading the host immune response, replicating the genomic RNA, and producing the subgenomic mRNAs that encode the structural proteins. NSPs 1–10 are produced from Orf1a as the self-cleaving polypeptide pp1a.^13,16^ As the ribosome approaches the stop codon of Orf1a, the programmed frameshifting element pseudoknot can cause the ribosome to slip backward by one position.^16−18^ If frameshifting occurs the ribosome will continue translating into Orf1b, producing pp1ab, which comprises NSPs 1–16. Known as the golden mean hypothesis of ribosomal frameshifting, incorrect stoichiometry of early replication products, in this case pp1a and pp1ab, disrupts the replication cycle and reduces virus propagation.^4,13,19^

The basic mechanism of −1 ribosomal frameshifting is known, although there are many levels of regulation at play that are not understood.^17,20−22,46^ Generally, a structured region of the RNA causes a translating ribosome to pause over a so-called slippery site with a nucleotide sequence pattern of X XXY YYZ composition.^16,18,23,24^ This structured region is most often a pseudoknot, which forms 6–8 nts downstream of the slippery site. The pseudoknot structure opposes the translocating ribosome, which creates tension that causes the slippery site codon interactions with P- and A-site tRNAs to slide backward by one nucleotide from X XXY YYZ to XXX YYY Z, resulting in a −1 shift in the reading frame.^16,18,23−25^

When incorporated into luciferase reporter mRNA constructs, the minimal sequence of frameshifting pseudoknots from different viruses induces frameshifting at an internally consistent frequency, but this frequency varies widely from virus to virus.^27^ For example, the programmed frameshifting element from West Nile virus induces frameshifting around 70 to 80% of the time, while the SARS-CoV-1 PFSE induces frameshifting around 15 to 30% of the time.^5,27,28^ Force extension curves of the different pseudoknots in an optical trap reveal that those elements populating more conformations induce frameshifting more frequently.^5,27,28^ A linear correlation between the rate of frameshifting *in vitro* and the number of conformations a pseudoknot can adopt can be drawn using the calculated Shannon entropy of each pseudoknot.^29^ The SARS-CoV-1 and SARS-CoV-2 PFSEs adopt only two conformations under tension, consistent with other pseudoknots that induce comparable rates of frameshifting.^5,28,29,50^ The conformational dynamics of frameshifting pseudoknots are clearly one important component of this highly regulated mechanism. An experimentally determined tertiary structure may help to illuminate how these conformations contribute to the regulation of frameshifting.

In SARS-CoV-2, the PFSE encompasses the roughly 80-nt-long sequence (residues 13462–13542 in the HB01 strain, gen bank number NC_45512.2) that includes a hepta-nucleotide slippery site, nucleotides 13462–13468 in the genomic numbering, and a spacer region followed by the proposed RNA pseudoknot structure in positions 13474–13542 in the genome (Figure 1A).^30^ Interestingly, this pseudoknot sequence is nearly perfectly conserved from SARS-CoV-1, the etiological agent of the 2003 coronavirus pandemic, with the exception of a single point mutation at position C 13444 in SARS-CoV-1 (Gen bank number AY278488.2) which corresponds to an A in position 13533 in SARS-CoV-2.^12,33,59^ The pseudoknot structure of the frameshifting element from SARS-CoV-1 has been well-characterized previously by chemical probing, mutational, and NMR studies of minimal constructs.^12,14,21^ The secondary structure of the SARS-CoV-1 PFSE derived from these studies (Figure 1B) has been used to guide molecular dynamics and tertiary structure prediction models.^8−10,32^ Briefly, the PFSE’s secondary structure comprises stem 1 (labeled S1 in Figure 1) at its 5′ end, which leads into loop 1 (L1) followed by stem 2 (S2; Figure 1B). Stem 3 (S3) forms below stem 1 and folds the RNA structure back on itself by forming the 12-nucleotide long loop 2. Stem 3 leads into loop 3 (L3), which spans the gap between the end of stem 3 and the start of stem 2. Stem 2 forms the long-distance interactions in the pseudoknot, which defines it as an H-type, and ties the 5′ end of the RNA to the 3′ end (Figure 1B).^12,31^

Despite the one nucleotide substitution, the SARS-CoV-2 PFSE likely adopts the same conformation as the SARS-CoV-1 PFSE. Computational modeling predicts similar structures for both sequences, and NMR studies show close agreement with the predicted three stem structure in both cases.^8−10,31,33−35^ Small-angle X-ray scattering diffraction analysis by Kelly et al. has shown that the SARS-CoV-1 frameshifting pseudoknot and the SARS-CoV-2 frameshifting pseudoknot have nearly identical topology.^33^ Functionally, both SARS-CoV-1 and SARS-CoV-2 pseudoknots frameshift to appreciable rates *in vitro* and in cells.^33^ Likewise, in both cases, frameshifting is inhibited in the presence of the ligand MTDB, which implies that both PFSEs bind the ligand.^7,33^ This evidence allows us to apply the established research on the SARS-CoV-1 PFSE to this new SARS-CoV-2 PFSE in our investigation of the structure.

To contribute higher resolution information about the SARS-CoV-2 PFSE structure, we have applied the chaperone assisted RNA crystallography method to the PFSE. We created a modified construct of the PFSE lacking the upstream slippery site and spacer region. This structured RNA was then bound to an engineered humanized murine antibody fragment to facilitate cocrystallization. Using iridium hexammine, we solved the structure using a combination of single angle anomalous diffraction phasing and molecular replacement. Here, we report the structure of the SARS-CoV-2 programmed frameshifting element pseudoknot solved by X-ray diffraction to 2.09 Å.

## Results

### Design and Characterization of Crystallization Construct

The PFSE region comprises the so-called slippery site (13462–13468), followed by a spacer region and finally the core pseudoknot spanning nucleotides 13474 to 13542 in the genomic numbering for strain HB01 (Figure 1A).^12,15,30^ Our crystallization construct includes only the minimal sequence for the pseudoknot (13474–13541). We omitted the spacer and the slippery site sequences from the crystallization construct due to the high flexibility expected for single-stranded regions at the ends of RNAs, which can reduce crystallization efficiency. In this construct, the starting residue, nucleotide 13474, was mutated from U to G to enhance *in vitro* transcription efficiency. This residue is predicted to be unpaired and is not considered essential for pseudoknot formation. It was unclear whether the final uridine of the pseudoknot sequence (residue 13542) would be paired.^9^ To avoid a dangling nucleotide in the construct, which can compromise the crystallization construct rigidity, this nucleotide was excluded from the template sequence. However, upon sequencing RNA extracted directly from the crystal, we found that the construct that crystallized had an untemplated G added to the 3′ end (see below and Supplementary Figure S3). We refer to this minimal version of the PFSE as the wild type PFSE construct.

RNA elements often resist forming well-diffracting crystals due to their biophysical properties such as instability, negatively charged backbone, conformational heterogeneity, and limited functional group diversity for mediating specific lattice contacts. Our lab has found that antibody fragment (Fab) RNA complexes crystallize more readily than RNA alone. In previous work, we reported developing a suite of humanized murine antibody fragments and their cognate RNA motifs, which can be grafted into structured RNA targets to create a Fab binding site.^36,37^ Among these, Fab BL3–6 binds to hairpins with the loop sequence (AAACA) closed by a GC pair, which we call the BL3–6-binding epitope. The most suitable location to graft the BL3–6-binding epitope into the PFSE was loop 2, at the base of stem 3. The SARS-CoV-1 PFSE has been shown to retain wild type levels of frameshifting activity *in vitro* when loop 2 was replaced with a GUUG tetraloop; therefore we expected that the PFSE structure could tolerate an AAACA pentaloop in the loop 2 position.^14^ We refer to this Fab-binding PFSE construct as PFSE BL3–6. In an electrophoretic mobility shift assay (EMSA), the PFSE BL3–6 construct formed a mobility shifted species in the presence of Fab BL3–6, suggesting that the grafted sequence formed the expected loop and had not altered the predicted RNA secondary structure as shown in Supporting Informaiton Figure 4 (Figure S4).

### Crystallization and Structure Determination

RNA, in the complex with an antibody fragment, was concentrated to 6 mg mL^–1^ and crystallized in 2% v/v tacsimate at pH 4, 0.1 M sodium acetate trihydrate at pH 4.6, and 16% w/v polyethylene glycol 3,350 (which has a final a measured pH of 4.8) and was further optimized with the addition of either 0.01 M sarcosine or 0.01 M betaine hydrochloride. Some crystals were soaked in cryogenic protectant containing iridium hexammine to aid in phasing using anomalous dispersion. These crystals, with and without heavy metal soaking, yielded multiple data sets with the best diffracting to 2.09 Å. To solve the structure, data sets with a strong anomalous signal and low resolution were phased in Phenix using a combination of single-wavelength anomalous diffraction (SAD) experimental phasing and molecular replacement using the Fab BL3–6 model.^36,38−40^ An initial electron density map was generated, and a partial model of the RNA was then built in COOT.^41^ This partial RNA model and the Fab model were then used to find a molecular replacement solution for a separate high resolution native diffraction data set. This solution gave complete electron density maps. Iterative rounds of building and refinement in this high-resolution data set yielded a complete structure for the PFSE pseudoknot at 2.09 Å with an *R*_work_ of 19.93% and an *R*_free_ of 23.08% (additional statistics reported in Table 1). The coordinates for this structure have been deposited in the PDB under accession code 7MLX.

**Table 1:** X-ray Crystallography Data Collection and Refinement Statistics

data collection	
space group	C 2 2 21
resolution range	67.71–2.09 (2.17–2.09)
cell dimensions	
*a*, *b*, *c* (Å)	76.83, 143.24, 133.64
α, β, γ (deg)	90, 90, 90
R-merge	0.083 (0.730)
I/ σ (I)	15.98 (2.50)
C*C*_1/2_	0.99 (0.80)
CC*	1 (0.94)
CC work/CC free	0.95(0.87)/0.88 (0.78)
completeness (%)	99.89 (99.93)
multiplicity	6.8 (6.7)
refinement	
no. unique reflections	43945 (4321)
*R*_work_/*R*_free_ (%)	0.1993 (0.2550)/0.2308 (0.2886)
RMS deviations	
bond lengths (Å)	0.007
bond angles (deg)	0.97
average B-factor all atoms (Å^2^)	40.78
Ramachandran plot of all protein residues	
favored (%)	97.64
allowed (%)	2.36
number of residues	
RNA	65
protein residues	430

### The Global Structure Reveals a Three-Stemmed Pseudoknot Matching Previous Predictions

The SARS-CoV-2 PFSE forms a three-stemmed H-type pseudoknot structure with three loops, consistent with original predictions for the SARS-CoV-1 PFSE by Plant et al. and confirmed as the general secondary structure for the SARS-CoV-2 PFSE more recently.^12,14,31^ In our structure (Figure 1C and D), stem 1, shown in green in all figures, begins at the 5′ end and forms between nucleotides 13474 and 13484 on the 5′-side and nucleotides 13493 and 13504 on the 3′-side (Figure 1C). Interestingly, the 5′ end of stem 1 threads through the ring created by loop 3 (Figure 4E and Figure S2), a feature inferred from midresolution cryo-EM models (see discussion).^4,43^ The 5′ strand of stem 1 leads into loop 1, colored cyan in all figures, which encompasses nucleotides 13485–13488. Within loop 1, nucleotides 13485 and 13486 form interactions with nucleotides 13494 and 13493 of stem 1, respectively. The loop 1 interactions position G13489 for pairing with the 3′ end of the RNA to form stem 2, shown in navy in all figures. Stem 2 is G-rich and comprises nucleotides 13489–13492 pairing with nucleotides 13541–13537, respectively. The 5′ end of nucleotide 13538 connects to loop 3, shown in magenta or gray when residues lack electron density. Loop 3 encompasses nucleotides 13533–13537 and connects the end of stem 2 with stem 3. Stem 3, colored orange in all figures, coaxially stacks below stem 1. Stem 3 encompasses nucleotides 13505–13513, which pair with nucleotides 13523 to 13532 with residue 13526 unpaired. This helically stacked three-stemmed structure approximately matches the predicted secondary structure of the PFSE (Figure 1B).^12^ Nevertheless, we observe several base pairing differences, detectable because of the high-resolution data.

### Loop 1 Organization Facilitates Formation of the Pseudoknot in Stem 2

**Figure 2:**
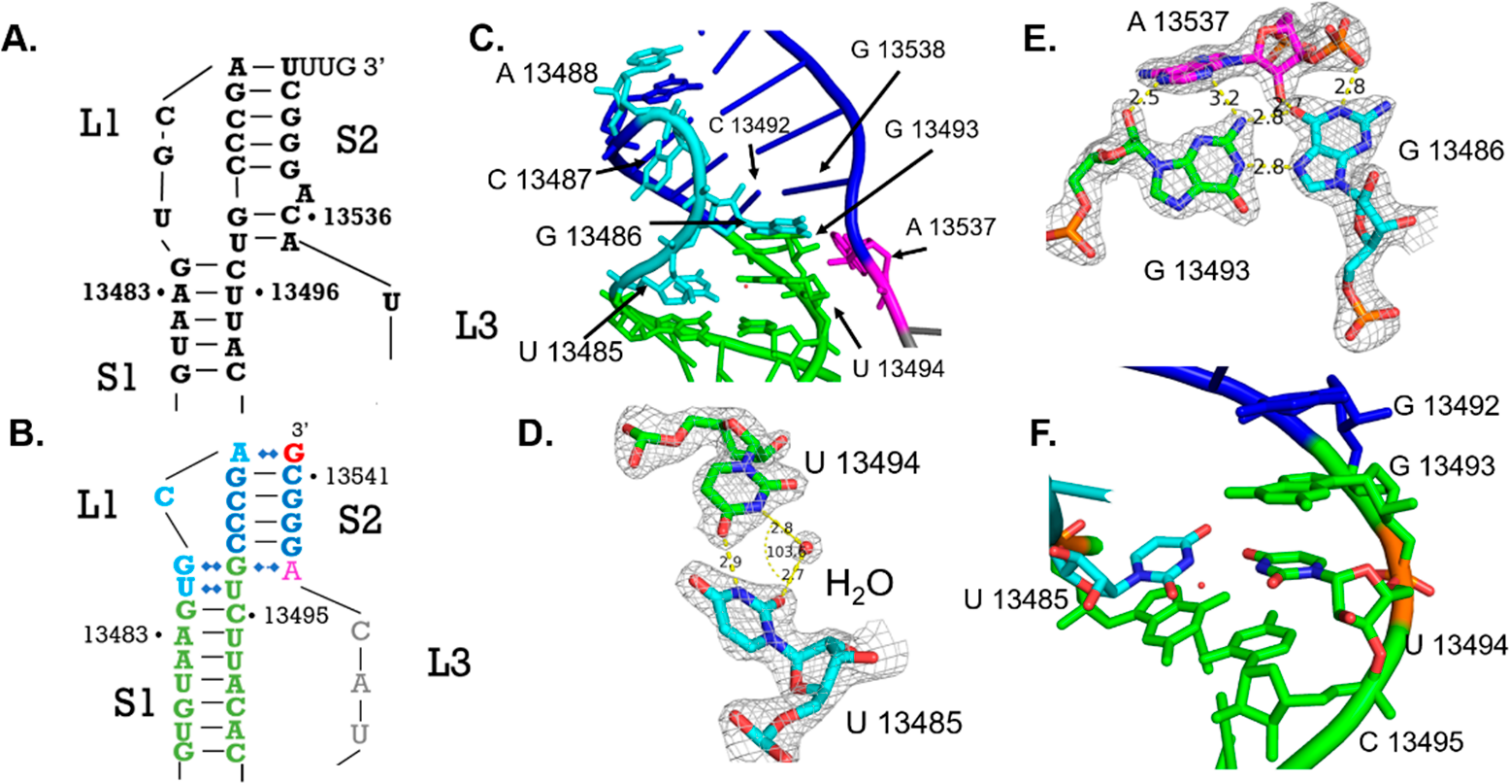
Organization of loop 1 (indicated as L1 in figures). (A) Predicted secondary structure interactions of loop 1; some secondary structure models predict that A13488 forms a base pair with U13541.^9,12^ (B) Crystal-structure-derived secondary structure of loop 1 colored cyan as defined in Figure 1. (C) Crystal structure model of loop 1 (cyan). (D) Noncanonical pairing between U13485 (cyan) and U13494 (green) mediated by an ordered water molecule. (E) G13486–G13493–A13537 base triple at the stem 1 (green)—stem 2 (blue) junction. G13486 (cyan) from L1 interacts with the phosphate oxygen of A13537 (magenta) and forms a Hoogsteen base pair with G13493 (green). (F) Base stacking interactions holding U13495 in the stem 1 helical stack.

In the helically stacked conformation of the PFSE, loop 1 forms interactions that position the descending strand (in our illustrations) of stem 2 to form the long-distance H-type pseudoknot interaction. Starting from the 5′ end of loop 1, nucleotide U13485, which was predicted to be unpaired (Figure 2A), forms a water-mediated base-pairing interaction with U13494 in stem 1 involving the O2 keto group and N3 imino group, respectively (Figure 2D). Additionally, the N3 imino NH of U13485 donates a hydrogen bond to the O4 keto group of U13494. While U13485 was predicted to be unpaired, nucleotide U13494 was predicted to pair with A13535 as part of stem 2. However, in our structure, U13494 faces inward toward stem 1 and is sandwiched in place by base stacking interactions with G13493 and C13495 (Figure 2F). The base pairing between U13494 and U13485 keeps the loop 1 strand close to the core of stem 1, which helps position G13486 to form its interactions.

**Figure 3:**
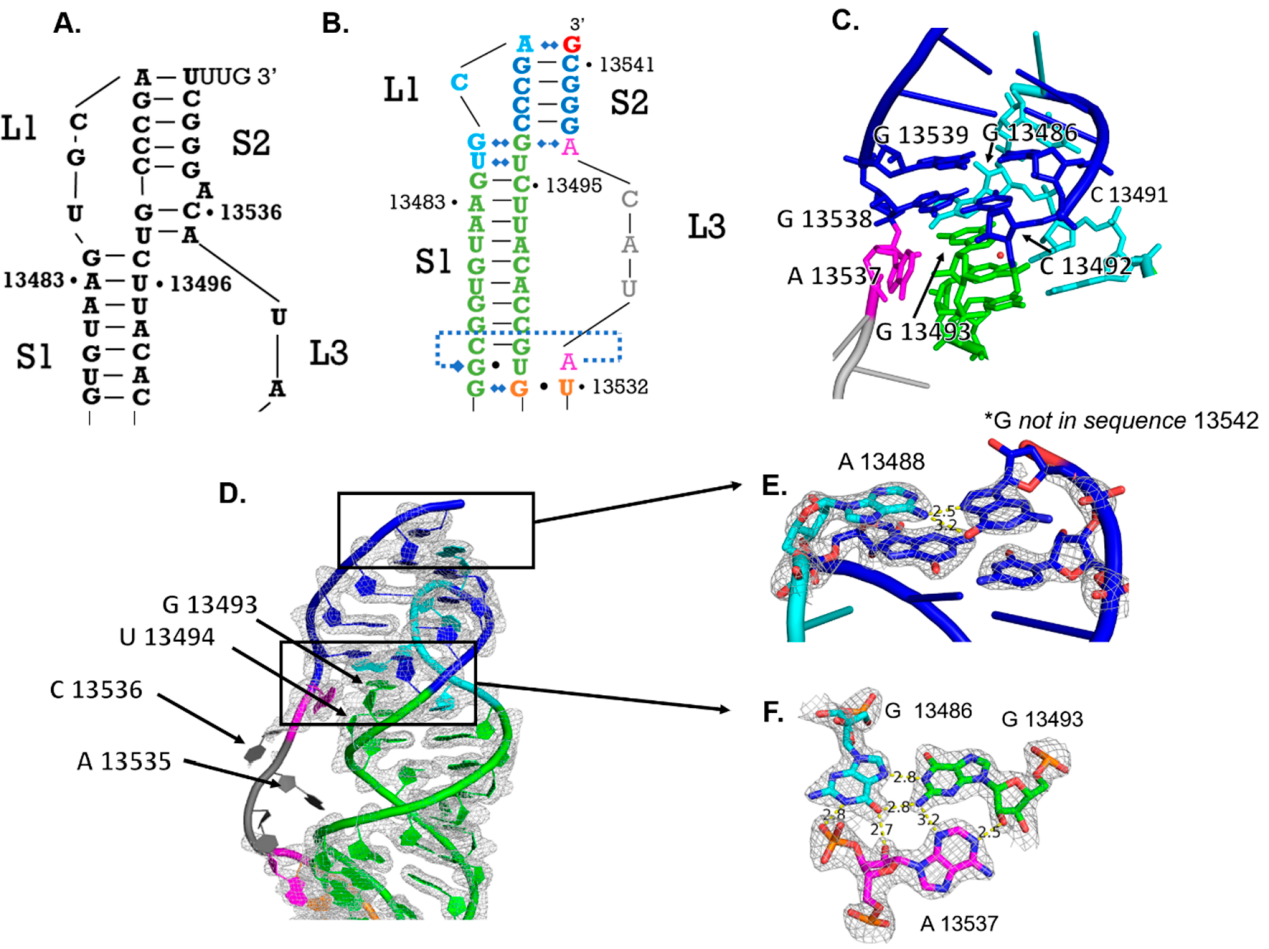
Stem 2 (labeled S2 and colored navy) and loop 3 (labeled L3 colored magenta for residues with density and gray for residues without density) differ from predictions. (A) Predicted secondary structure of stem 2.^12^ (B) Secondary structure derived from the crystal structure. (C) Stem 2/stem 1 junction showing the inflection point at G13493 and C13492. (D) Crystal structure displaying electron density for stem 2 and loop 3; electron density map displayed as mesh. (E) A13488–G13542 Hoogsteen interaction with the electron density map displayed as mesh. (F) A13537, G13493, and G13486 base triple with electron density map displayed as a mesh.

G13486 engages in a base triple interaction with G13493 and A13537. G13486 uses its Hoogsteen face to interact with the Watson–Crick face of G13493 and uses its exocyclic keto group to accept a hydrogen bond from A13537’s 2′ hydroxyl group (Figure 2E and Figure 3F). We consider this G13486–G13493–A13537 base triple the transition between stem 1 and stem 2 because G13486 is no longer stacking on the stem 1 helical axis. Above this point, C13487 twists away from G13486, leaving C13492 to base pair with G13538 (Figure 2C). C13487’s nucleobase plane has rotated relative to the base planes of G13486 and A13488 to run parallel to the helical axis of stem 1 (Figure 2C). After the turn at C13487, the ribose-phosphate backbone reverses direction again to allow A13488 to engage in a noncanonical pairing interaction with the 3′-terminal G13542, forming the uppermost base pair of the pseudoknot duplex, stem 2. This positions the nucleotides that follow, G13489 and C13490–C13492, to base pair with the opposing G-rich strand at the 3′ end of the RNA, G13538–13540 and C13541.

### Stem 2 Is Shorter than Predicted

Stem 2 was originally predicted to consist of six base pairs between nucleotides 13488–13494 and nucleotides 13542–13535 with A13537 bulged out from the helix (Figure 3A). However, our structure shows that stem 2 consists of Watson–Crick base pairs between nucleotides 13488–13492 and nucleotides 13542–13538 (Figure 3B), with A13488’s Watson–Crick face forming a Hoogsteen interaction with G13542 and capping the helix from the top (Figure 3E). Residue A13537 ends the stem 2 stack and forms an A-minor interaction with the aforementioned G13486–G13493 Hoogsteen pair at the base of stem 2. In this base triple, A13537 forms two hydrogen bonds: one involves its N6 amine and the 2′ OH of G13493, and the other involves N3 and the exocyclic amine of G13493 (Figure 3F). A hydrogen bond between G13538’s nonbridging phosphate oxygen and the imino NH of G13486 further stabilizes the base triple, in addition to the 2′ OH and G13486 keto group hydrogen bond mentioned previously (Figure 2E and F). A key difference between the predicted stem 2 and the crystal structure is that nucleotides C13536 and A13535 do not form the predicted base pairs with G13493 and U13494, respectively. Instead, there is clear density showing that G13493 and U13494 direct their Watson–Crick faces away from the central axis of stem 2, leaving C13536 and A13535, which lack clear density, without their predicted pairing partners (Figure 3D). We conclude that, in this conformation, stem 2 is shorter than predicted.

Although the DNA template used for transcription of the PFSE construct was designed to terminate transcription at nucleotide 13541, the electron density map showed density for an additional 65th nucleotide. As T7 RNA polymerase is known to add untemplated nucleotides to the 3′ terminus of transcripts, we used a simple method to sequence the 3′ ends of *in vitro* transcribed RNAs extracted from crystals to confirm the presence of an additional nucleotide and reveal its identity.^42^ Briefly, the RNA was polyadenylated to create a primer binding site for a poly(T) reverse primer for reverse transcription into cDNA. The resulting cDNA was amplified and sequenced. This not only revealed the identity of the terminal residues but also quantitatively measured the enrichment for each of the four nucleotides randomly added by T7. RNA extracted from washed crystals shows a G predominating after C13541 (Figure S3). By contrast the mother liquor contains transcripts with a mix of G and C (Figure S3). The enrichment for G over C in the crystal may be due to the crystal contact involving stacking of G13542 with a mirrored symmetry mate of the RNA. Whether the native U in position 13542 forms a base pair with A13488 remains unclear.^9^

### The C56A Point Mutation between SARS-CoV-1 to SARS-CoV-2 PFSEs Forms a Base Triple

**Figure 4:**
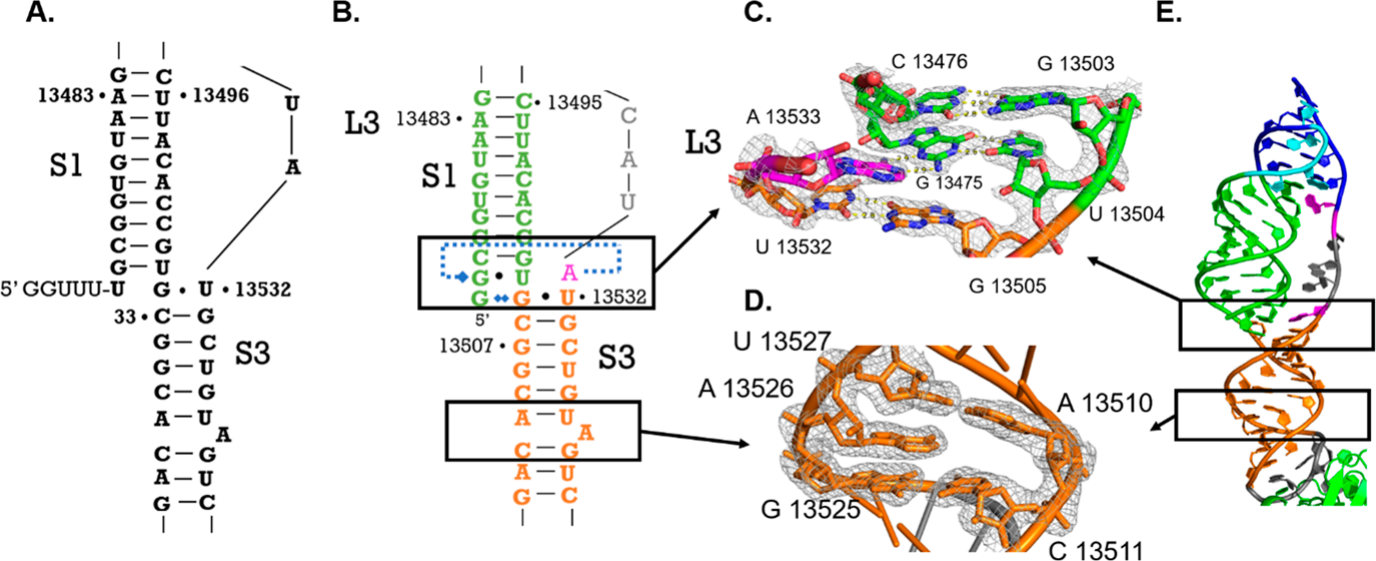
Stem 1 and stem 3 junction. (A) Predicted secondary structure of the stem 1/stem 3 junction.^12^ (B) Crystal structure derived secondary structure of the stem 1/stem 3 junction. (C) Stem 1/stem 3 base triple formed between A13533 (pink), G13475 (green), and U13504(green). G13505–U13532 (orange) wobble pair that ends the stem 3 helical stack. C13476–G13503 (green) base pair illustrating the start of the stem 1 helical stack. Electron density for this region displayed as mesh. (D) Unpaired nucleotide A13526 does not bulge from the helix as predicted and resides within the helix via stacking interactions with nucleotides U13527 and G13525; electron density displayed as mesh. (E) Locations of these regions in the PFSE structure.

Secondary structure predictions and computational models of the PFSE vary in the arrangement of A13533.^9,10,34,35^ This residue is often modeled as unpaired in Loop 3 or paired to U13504 in Stem 3. However, in this structure we see clear density for a triple base pair involving G13475, U13504 and A13533 at the S1/S3 junction (Figure 4C). Stem 3 ends at a wobble base pair between G13505 and U13532; above this, A13533 breaks away from this helical stack to form a Watson–Crick sugar-edge interaction with G13475, which forms a wobble pair with U13504 (Figure 4C and E). This base triple interaction may help stabilize the vertical arrangement of stem 1/stem 3.

Compared to the PFSE of SARS-CoV-1, the PFSE of SARS-CoV-2 has a single C to A substitution at residue 13533 in SARS-CoV-2 which corresponds to 13444 in SARS-CoV-1. The C to A mutation itself seems to have no effect on frameshifting efficiency as both frameshift to appreciable extents, although the in vitro and in vivo efficiencies vary significantly.^33^ When position 13533 is modeled as a C in our structure, we see that the guanidinium group is poised to make the same Watson–Crick sugar edge hydrogen bonding interactions that the guanidinium group of A makes (Figure S1). Thus, the SARS-CoV-1 PFSE also appears capable of forming a triple base interaction at the S1/S3 junction, supporting the functional relevance of the base triple.

The S1/S3 junction is also the site of the 5′ end threading though the ring created by stem 1 and loop 3, consistent with observations from other structural studies of the PFSE.^4,43^ Our structure shows that threading is due to the twist of the stem 1 helix holding the first five nucleotides in place as loop 3 and the 3′ end of the RNA fold over top (Figure S2). Here, we also find that G13475, included at the 5′ end for transcriptional efficiency in place of the native U, interacts with the G13505–U13532 wobble pair of stem 3 (Figure S2). In this base triple, the N1H imino group and N2H_2_ exocyclic amine of G13474 donate hydrogen bonds to N7 and the O6 keto group of G13505, respectively (Figure S2). The native U nucleotide in position 13474 would not be able to make the analogous interactions. While the non-native interaction of G13474 may stabilize the vertical conformation, its absence in the native structure would not preclude formation of the vertical conformation.

### Geometric Constraints Restrict the Conformation of Nucleotides with Missing Density

Loop 3, which connects stems 2 and 3, consists of nucleotides 13533, 13534, 13535, 13536, and 13537 in our structure. Density for residues 13534, 13535, and 13536 is missing from the data sets, suggesting this loop is flexible and not well organized in the crystal (Figure 3D). Nevertheless, the 3′ end of A13533 and the 5′ phosphate of A13537, which are well-defined by the electron density, are separated by 17.4 Å. The length of one fully extended nucleotide of ssDNA is 6.7 Å.^44^ Single-stranded RNA is expected to be more compact than ssDNA due to steric restraints imposed by the 2′ OH on the sugar pucker conformations. If nucleotides 13534, 13535, and 13536 in loop 3 were fully extended, they would span a maximum distance of 20.1 Å, leaving only ∼2.7 Å of slack for bending. In a study of a 40-nucleotide strand of poly(U) RNA, a total contour length of 196 Å was measured, giving 4.9 Å as the average length of each nt.^44^ In a relaxed state, we would therefore expect three nucleotides to span 14.3 Å, meaning that in spanning 17 Å the nucleotides in loop 3 are likely extended and less flexible than they would be if their ends were free. We anticipate that the residues in this region likely possess enough flexibility to wiggle back and forth like a short string held at both ends but must maintain a relatively extended backbone conformation to bridge the distance between residues 13533 and 13537.

### Stem 3 Organization Matches Predictions

The base pairing for stem 3 observed in our structure agrees with the predicted pairing. In previous work, A13526 frequently exhibits sensitivity to chemical modification indicative of a single-stranded nucleotide.^14^ We find this residue unpaired and stacked within stem 3 (Figure 4D). Previous mutational studies have shown that deletion of A13526 or insertion of a corresponding U to form a base pair both reduce frameshifting efficiency in the SARS-CoV-1 PFSE, suggesting that this residue’s unpaired state contributes to the frameshifting mechanism. Nevertheless, complete deletion of stem 3 rescues frameshifting.^14^ Often computational modeling predicts that the PFSE is bent at the junction between stem 1 and stem 3, and cryo-EM structures exhibit this bend.^4,8−10,32,35,43^ Having A13526 unpaired could contribute to the dynamic character of stem 3, facilitating sampling of the bent conformation.

## Discussion

### Comparing Existing Structural Probing Data with Our Crystal Structure

**Figure 5:**
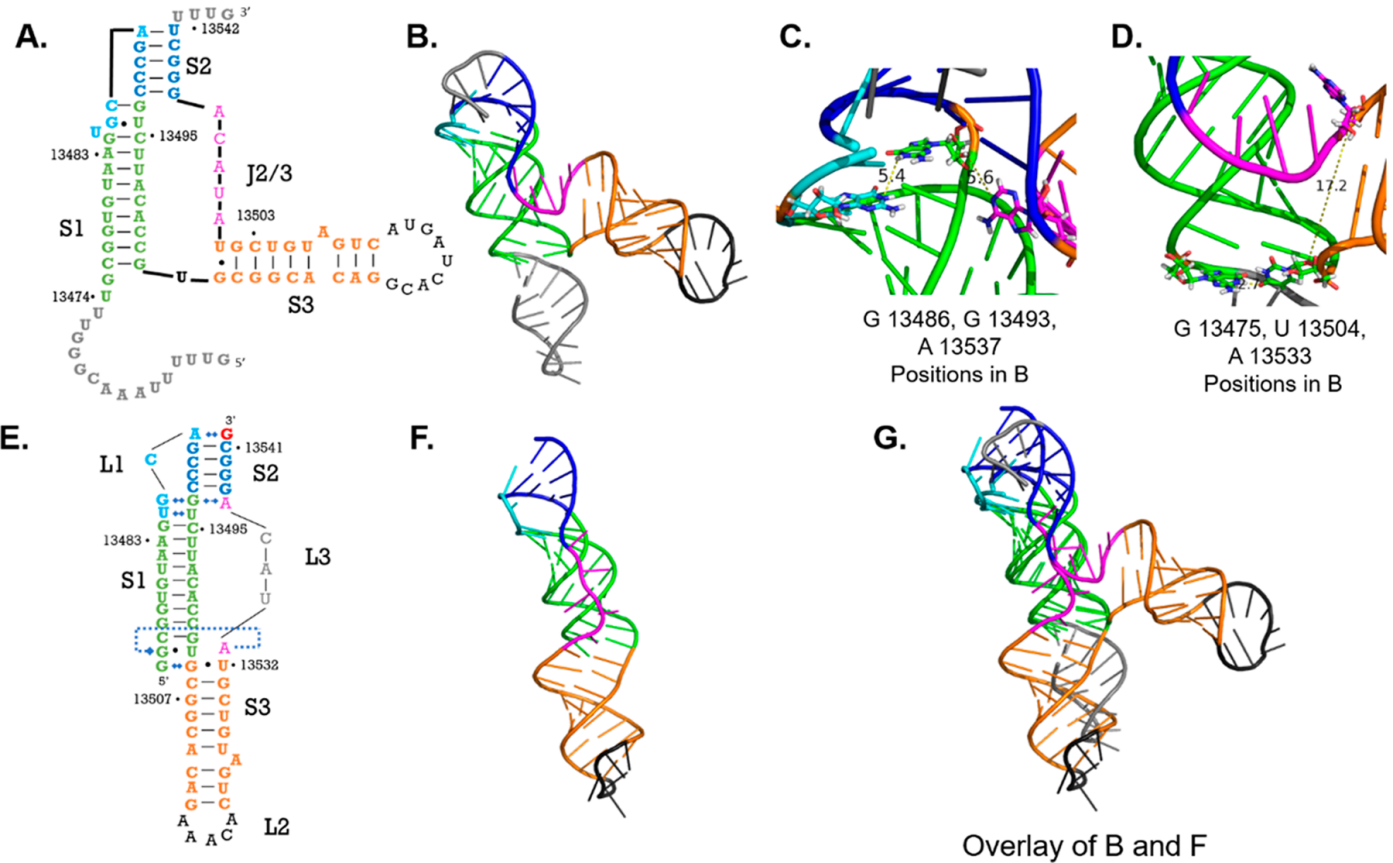
SARS-CoV-2 PFSE cryo-EM structure solved by Zhang et al.^43^ overlaid with crystal structure reported here. (A) Secondary structure model Zhang et al.^43^ cryo-EM reported renumbered to correspond to our numbering for comparison; stem 1 colored green; loop 1 colored cyan; stem 2 colored navy; loop 3 colored magenta; stem 3 colored orange; loop 2 colored black; nucleotides present in this cryo-EM structure but not in our structure are colored gray. (B) cryo-EM model (PDB code: 6XRZ) displayed as a cartoon colored as in A. (C) Stem 1/stem 2 base triple residues, G13486, G13493, and A13537, displayed as sticks in the cryo-EM structure; measurements display distance between atoms that interact in the crystal structure. (D) Stem 1/stem 3 base triple residues G13475, U13504, and A13533 displayed as sticks in cryo-EM structure. (E) Secondary structure map derived from the crystal structure colored to match A. (F) Crystal structure displayed as a cartoon colored as in B. (G) Overlay of cryo-EM model (B) and our crystal structure (F).

It is possible that our Fab-hairpin crystallization module and crystal packing forces facilitated formation of the linear conformation we observe in this crystal structure. However, at a minimum, the secondary structure shows close agreement with the base pairing pattern inferred from chemical probing, mutational analysis, and NMR.^11,34,35,43,45^ Nevertheless, the biological relevance of the linear conformation and associated base triples observed in this high-resolution structure await further investigation either in the context of frameshifting or in another stage of the viral lifecycle. We note that alternate orientations of stem 3 relative to stem 1 and stem 2 do not seem to require major rearrangements to the secondary structure as illustrated in Figure 5, suggesting that the PFSE potentially samples the linear conformation in solution.

Analysis of the SARS-CoV-1 PFSE by chemical and enzymatic probing and NMR, supported by mutational studies, indicate that frameshifting depends on the PFSE adopting a three stemmed H-type pseudoknot-like structure.^12,14,31^ Recent DMS and SHAPE probing performed on minimal constructs of the highly homologous SARS-CoV-2 PFSE implicate an analogous three stemmed structure.^11,34,35,43,45^ NMR analysis detected base pairing interactions consistent with the same three stemmed arrangement of the SARS-CoV-2 PFSE in solution as well.^31^ SAX data collected on the SARS-CoV-1 and SARS-CoV-2 PFSE in combination with in vitro translation assays in the presence and absence of the frameshifting inhibitory ligand MTDB all support the conclusion that these SARS-CoVs’ PFSEs adopt the same structure and perform frameshifting through the same mechanism.^5,33^

This first experimentally determined high resolution structure of a SARS-CoV PFSE has revealed interactions that were not identified via chemical probing and NMR assays previously applied to CoV-1 or CoV-2 PFSE. These new interactions provide a structural hypothesis that may explain the C to A variation at residue 13533 in SARS-CoV-2 and 13444 in SARS-CoV-1, which are otherwise absolutely conserved within the PFSE. While the secondary structure closely matches predictions for both SARS-CoV-1 and SARS-CoV-2 PFSEs, the base triple interactions detected at the S1/S2 and S1/S3 junctions in our 2.09 Å resolution data set may contribute to conformational sampling, a process critical for frameshifting in cells, or influence interactions with cellular factors.^27,29^ The roles that the vertical conformation and the accompanying base triples play in the mechanism of frameshifting can now be investigated with directed tests informed by the structure.

The PFSE region may only sample this three stemmed arrangement occasionally, as the *in vivo* probing reactivities of the PFSE in its genomic context exhibit differences compared to *in vitro* chemical probing reactivities of minimal constructs.^11,34,43,47^ Lan et al. showed that the *in vitro* DMS reactivities of the PFSE become more similar to those observed *in vivo* as the construct is elongated to include more of the genomic sequence.^11^ This suggests that the PFSE region likely samples different or additional conformations in its genomic context, potentially forming long distance interactions. However, during translation with translocating ribosomes upstream and downstream, the PFSE region would be unfolded and isolated from the rest of the genomic RNA enabling formation of the frameshifting structure.^4,17,46^ Additionally, the finding that the MTDB can inhibit viral replication and alters the ratios of the conformations the PFSE can adopt suggests that structures adopted by the minimal PFSE element have relevance for viral function.^4,5^

### Comparison of our Crystal Structure to cryo-EM Structures

Currently, two midresolution cryo-EM models exist for the PFSE. Both share important features with our model, and the differences may provide hints as to the mechanism of ribosomal frameshifting. Zhang et al. used cryo-EM data at 6.7–6.9 Å resolution as a constraint for the Ribosolve pipeline to model the structure of the PFSE and slippery site (nt 13 459–13 548 in NC.045512.2).^43^ The second cryo-EM structure was reported by Bhatt et al.^4^ In this case, the PFSE pseudoknot is present in the context of the genomic mRNA encoding NSP 10–12 bound to a ribosome paused over a mutated slippery site.^4^ Here the PFSE region is solved to 5–7 Å.^4^ The coordinates for the ribosome bound structure have not yet been deposited, so making direct comparisons between this structure and our crystal structure is not yet possible.

Both cryo-EM structures find the PFSE in a “bent” arrangement, where stem 1 and stem 2 are helically stacked while stem 3 bends perpendicularly away from stem 1.^4,43^Figure 5 illustrates the similarities and differences between the Zhang et al. structure (PDB code: 6XRZ) and our crystal structure.^43^ Our 2.09 Å structure confirms many of the base pairing interactions that remained ambiguous at 6.7 Å. The secondary structures reported by each work identify the same differences from the “literature” secondary structure as we have found. The structures agree that stem 2 ends at A13537, and the crystal structure reveals additional details about the specific base pairing interactions at the S1/S2 helical junction. Our structure also shows the same 5′ end threaded topology first identified in these cryo-EM structures. In contrast to the cryo-EM structures, our crystal structure adopts a vertically stacked conformation, which has been consistently predicted by computational modeling but not yet observed experimentally.^9,10,34^ This structure could represent the second SARS-CoV-2 PFSE conformation.

Curiously, neither of the base triple pairing interactions we report are present in the cryo-EM models, although this may be due to the limited resolution of these structures. The bent conformation of the PFSE and slippery site observed in the solution cryo-EM model (PDB code: 6XRZ) positions the S1/S2 base triple nucleotides (G13486, G13493, and A13537) too far apart to form the base triple (Figure 5C).^43^ Additionally, in the ribosome-bound structure, G13486 forms direct interactions with the N-terminal domain of US3 while A13537 remains unpaired in the J2/3 region.^4^ Therefore, the S1/S2 base triple we observe would have to be dissolved in the bent conformation and in the ribosome bound state. Interestingly, mutations to G13486 or A13537 in isolation were shown to reduce frameshifting frequency markedly, although the role of A13537 is unknown.^4^ The PFSE’s intolerance to A13537 mutations combined with these structural observations suggest that G13486 may serve an additional structural role to orient G13493’s sugar edge to interact with A13533’s WC face. In other −1 frameshifting pseudoknots, elimination of known base triple interactions does reduce frameshifting efficiency by destabilizing the pseudoknot.^48^ These observations suggest that the S1/S2 base triple may act as a conformational switch or decision point between the bent conformation and the vertical conformation.

The base triple involving G13475, U13504, and A13542 at the S1/S3 junction may further stabilize the vertical conformation as it cannot be fully formed in the bent or ribosome-bound cryo-EM structures. In the ribosome bound structure, G13475, of the S1/S3 base triple, is inside the mRNA entry channel, but A13533 and U13504 are modeled as paired.^4^ In the cryo-EM structure of the element in isolation (PDB code: 6XRZ), G13475 and U13504 are close enough to base pair, while A13533 is more than 10 Å away (Figure 5D).^43^ Unfortunately, we lack mutational information for this region of the PFSE to illuminate the contributions of these nucleotides to frameshifting. In nature, however, the residue corresponding to A13533 in SARS-CoV-2 is a C (13444) in SARS-CoV-1. The role of this C to A substitution is not yet understood, but when A13533 is modeled as a C, the hydrogen bonding interactions with G13475 can still be formed (Figure S1).^33^ The role of this S1/S3 junction region now warrants deeper investigation as it could dictate a dynamical relationship between bent and linear conformations and thereby influence frameshifting efficiency.

Our crystal structure, the cryo-EM structure of the free PFSE with the slippery site, and the ribosome-bound PFSE-containing gRNA fragment have similar topologies and differ only at junctions where alternate pairing interactions or ribosomal interactions can occur. The crystal structure model represents a vertically stacked conformation, and the cryo-EM structures represent a bent or wedged conformation. Modeling of the PFSE by Omar et al. and Rangan et al. demonstrated that the arrangement of stem 1 and stem 2 relative to stem 3 can be flexible.^9,10^ Indeed, substantial data exist that support the hypothesis that the frameshifting element populates two distinct conformations that govern the efficiency of PFSE frameshifting.^27−29,50^

We caution, however, that conformational differences detected across the various models of the PFSE may be due to differences in RNA construct design and or ribosomal interactions. For example, our construct lacks the 5′ slippery site sequence, which forms a helix that coaxially stacks beneath stem 1 in the cryo-EM model. The slippery site helix could preclude formation of the vertically stacked conformation just as the ribosome does. Additionally, recent investigation of the SARS-CoV-2 RNA genome both within the cytoplasm and the virion infers entirely new secondary structures for the PFSE.^11,47,58^ Rather than frameshifting itself, the vertical conformation observed here could have significance for PFSE structural rearrangements that occur during other stages of the virial lifecycle. Moreover, as there are limited structures available for RNA pseudoknots, the high-resolution structure data presented herein have intrinsic value for the structure-based design of small molecule binders, as a starting point for molecular dynamics simulations, and as a framework for testing the relationship of the RNA structure to its function.

### Correspondence of the Structure with PFSE Dynamics and Folding Data

In programmed ribosomal −1 frameshifting pseudoknots, helical stacking is often a required feature to induce frameshifting, and tertiary interactions have been shown to stabilize frameshifting pseudoknots and promote efficiency.^48,49^ Frameshifting pseudoknots must also be conformationally dynamic to function, refolding into one among multiple conformations each time the ribosome reads through the mRNA.^28,49^ Optical tweezers can be used to both unfold the pseudoknot and mimic the tugging forces the ribosome would apply to a pseudoknot during translation.^17,29^ In reporter assays, the rate of frameshifting of any given pseudoknot correlates linearly with its conformational Shannon entropy, a statistical metric for conformational plasticity.^29^ Pseudoknots that occupy two conformations often induce frameshifting on the order of 20% efficiency.^27^ Nevertheless, frameshifting rates measured *in vitro* can be different than those measured *in vivo* or in infected cells due to additional interactions with proteins and the gRNA.

Unfolding force extension curves show that the SARS-CoV-2 PFSE can adopt two distinct conformations of roughly similar stability, falling in line with expectations given a frameshifting frequency in the 15–30% range.^50^ The more stable form (N) unfolds at an average force of 30 pN, and the slightly less stable form (N′) unfolds at an average force of 16 pN. Possibly, the two states correspond in part to the vertical and bent conformations, although we emphasize that this awaits careful validation. The base triples in the vertical conformation would be expected to provide additional stability and rigidity relative to a conformer lacking them. In line with this possibility, another H-type pseudoknot found in human telomerase RNA (ΔU177) was found to lose ∼16 pN of unfolding force upon disruption of two minor groove base triples, which is a similar difference in unfolding force between N and N′.^48,50^ It will be important to address whether the vertical conformation corresponds to one of the states in the unfolding experiments, and our structure will inform the design of atomic mutations for these tests.

## Methods

### Construct Design

Our crystallization target was based on nucleotides 13475–13541 in the SARS-CoV-2 genome, Gen Bank number NC_045512.2; these are the minimal residues predicted to form base pairs.^30^ We chose to exclude nucleotide 13542 in this crystallization construct because its binding to A13488 was unclear, and incorporating an unpaired nucleotide at the 5′ end could compromise the structural integrity of the crystallization complex. Typically, the PFSE is defined as this region as well as the 14 nucleotides upstream, which include the slippery site; these were excluded from the crystallization construct because long single-stranded regions of RNA can disrupt folding and crystallization of the RNA. This truncated construct we refer to as the wild type PFSE construct.

To enable binding to antibody fragment, Fab BL3–6, we mutated the nucleotide corresponding to loop 2 (13514 to 13522) to the sequence AAACA. This crystallization construct is referred to as the BL3–6 PFSE construct. We chose to mutate these residues because mutational studies have shown that loop 2 can be mutated to a common RNA tetraloop without altering the ratio of frameshifting in the SARS-CoV-1 PFSE, which is believed to be structurally identical to the SARS-CoV-2 PFSE.

V-fold^51^ predictions suggest that the BL3–6 PFSE does not contain non-native regions of complementarity that might be prone to disrupt the native secondary structure. A gel shift assay was performed on refolded PFSE RNA constructs and antibody fragment BL3–6 to show that the grafted nucleotides still form the expected solvent-exposed loop, and that Fab binding was only observed for the mutated construct.

### RNA Transcription and Purification

Single stranded DNA templates and primers for PCR and transcription were ordered from IDT encoding the transcription template for each RNA construct with a T7 promoter. Forward primers were ordered matching the T7 promoter region, and reverse primers contained a single 2′ O-methyl modification at the 3′ end to avoid untemplated additions by T7 polymerase.^52^ Transcription template DNA was amplified into double-stranded DNA using PCR. RNA was transcribed from the purified PCR product using an *in vitro* transcription reaction; 50 pmol mL^–1^ DNA template was incubated for 3 h at 37 °C in buffer containing 40 mM Tris-HCl, at pH 8.0, 2 mM spermidine, 10 mM NaCl, 25 mM MgCl_2_, 10 mM DTT, 40 U mL^–1^ RNase inhibitor, 5 U mL^–1^ thermostable inorganic pyrophosphatase, 5 mM of each NTP, and 50 μg mL^–1^ T7 RNA polymerase. Reactions were halted by the addition of RNase free DNAase1 at 5U mL^–1^ and incubation at 37 °C for 30 min. RNA was purified on a 10% denaturing polyacrylamide gel in 0.5 × TBE running buffer. The RNA was visualized with UV shadowing, extracted, and eluted into 10 mM Tris, at pH 8.0, 2 mM EDTA, and 300 mM NaCl buffer via overnight incubation at 4 °C. The eluted RNA was then concentrated and exchanged into double distilled H_2_O using a 10K Amicon filter and stored at −80 °C until further use.

### Fab Purification

The BL3–6 Fab expression vector (available upon request) was transformed into 55244 chemically competent cells (www.atcc.org) and grown on LB plates supplemented with carbenicillin at 100 μg mL^–1^. Nine colonies from the plates were chosen and inoculated to a starter culture with 100 μg mL^–1^ carbenicillin, which was grown at 30 °C for 8 h. Once the starter culture reached an OD 600 of 8, 15 mL of starter culture was used to inoculate 1 L of 2× YT media and grown for 24 h at 30 °C. The cells were then pelleted via centrifugation at RT, and the cell pellet was resuspended in 1 L of freshly prepared phosphate depleted media supplemented with 100 μg mL^–1^ carbenicillin. The cells were set to grow for 24 h at 30 °C, harvested via centrifugation at 4 °C, and frozen at −20 °C. Frozen cell pellets were lysed in PBS buffer supplemented with 0.4 mg mL^–1^ of lysozyme and 0.01 mg mL^–1^ of DNase I. After 30 min, PMSF was added to a final concentration of 0.5 mM. After 30 min, the cells were centrifuged for 45 min at 12 000 rpm and 4 °C. Lysate was transferred to new sterile bottles and centrifuged again for 15 min at 12 000 rpm and 4 °C. Supernatant was filtered through 0.45 μm filters into a sterile bottle (Millipore Sigma, www.sigmaaldrich.com), and Fab proteins were purified using the AKTAxpress fast protein liquid chromatography (FPLC) purification system (Amersham, www.gelifesciences.com) as described previously.^37^ The lysate in PBS buffer (pH 7.4) was loaded into a protein A column, and the eluted Fab in 1 M acetic acid was buffer exchanged back into the buffer PBS (pH 7.4) using a 30 kDa cutoff Amicon filter and loaded into a protein G column. The Fab was eluted from a protein G column in 0.1 M glycine (pH 2.7) and then buffer-exchanged into 50 mM NaOAc and 50 mM NaCl buffer (pH 5.5) and loaded into a heparin column. Finally, the eluted Fab in 50 mM NaOAc and 2 M NaCl (pH 5.5) was dialyzed back into 1× PBS (pH 7.4), concentrated, and analyzed with 12% SDS-PAGE using Coomassie Blue R-250 staining for visualization. Aliquots of Fab samples were tested for RNase activity using the RNaseAlert kit (Ambion, www.thermofisher.com). The aliquots of Fab samples were flash frozen in liquid nitrogen and stored at −80 °C until further use.

### Electrophoretic Mobility Shift Assay (EMSA)

To validate Fab binding to the BL3–6 PFSE RNA construct, purified RNA constructs in double distilled H_2_O were heated to 90 °C for 1 min, then cooled on ice for 2 min, then held at RT for 3 min. A refolding buffer (50 mM HEPES pH 8, 5 mM MgCl_2_, 100 mM KCl) was added, and the RNA was then incubated at 50 °C for 10 min to facilitate refolding. RNA was then mixed with either PBS as a negative control or a 1.1 M ratio of RNase–free Fab BL3–6 and incubated at RT for 30 min to establish equilibrium binding. Fab RNA complexes were separated by gel electrophoresis in a 12% polyacrylamide gel made in 0.5× TBE buffer supplemented with 5 mM MgCl_2_. The gel was stained with ethidium bromide and visualized via UV light and photographed (Figure S4).

### Sequencing Reactions

To determine the identity of the untemplated nucleotide observed in the electron density map, we sequenced the RNA from three sources: the transcription product, the mother liquor of the crystal drop, and the crystal itself. For the transcribed RNA, we followed the standard procedure for poly(A)-tailing (NEB) using 3 μg of RNA in reaction with *E. coli* poly(A) polymerase. For the mother-liquor-derived RNA samples, mother liquor was harvested from a 200 nL drop of crystallized complex. To ensure that all of the mother liquor solution was harvested, 0.5 μL of well solution was added to the drop prior to transferring the samples to an Eppendorf tube. For the crystal-derived RNA samples, after the mother liquor was harvested from the drop, the remaining crystals were washed three times with well solution. Then, the crystals were transferred to an eppendorf tube with 2 μL of RNase free water and crushed via pipetting. Samples with transcribed RNA and mother liquor- and crystal-derived RNA were denatured for 1 min in 90 °C. Then, they were cooled down on ice for 2 min and incubated for 3 min at RT. Denatured RNA samples were elongated with 1 μL of *E. coli* poly(A) polymerase 5000 U mL^–1^ (NEB), and 2 μL of 10× *E. coli* poly(A) polymerase reaction buffer with the addition of 2 μL of 10 mM ATP and RNase free water up to 20 μL as per the manufacturer’s instructions. The reaction was incubated at 37 °C for 30 min in a water bath. The reaction was halted by the addition of EDTA to a final concentration of 10 mM. RNA was ethanol precipitated and checked for poly(A) elongation on a 10% polyacrylamide gel stained with ethidium bromide. Polyadenylated RNA was used as the template in a reverse transcription reaction using SuperScript III (Invitrogen) according to the manufacturer’s instructions. cDNA was amplified using end specific primers and PCR with 30 cycles of amplification using an annealing temperature of 52 °C and *Taq* DNA polymerase (NEB). The double stranded DNA products were then submitted for sequencing. We note that this method is one of many commonly used to sequence the 3′ end of RNAs.^55−57^

### Crystallization

PFSE BL3–6 RNA was denatured in water by incubation at 90 °C for 1 min, in ice for 2 min, and at RT for 3 min. The RNA was then refolded by the addition of refolding buffer (50 mM HEPES at pH 8, 5 mM MgCl_2_, 100 mM KCl) and incubated at 50 °C for 10 min. Fab was added to the RNA at a 1:1.1 molar ratio of RNA/Fab and incubated at RT for 30 min before concentrating the complex to 6 mg mL^–1^ RNA via centrifugal filtration in a 10 kDa cutoff Amicon Centrifugal Filter Unit. Concentrated complexes were then filtered using 0.2 μm cutoff Millipore centrifugal filter units and used to set high-throughput hanging-drop vapor-diffusion crystallization screens at RT using commercially available screening kits from Hampton Research and Jena Bioscience using the Mosquito liquid handling robot (TTP Labtech). Crystals grew in 2% v/v tacsimate at pH 4. 0.1 M sodium acetate trihydrate at pH 4.6, and 16% w/v polyethylene glycol 3,350 and were further optimized with the addition of either 0.01 M sarcosine or 0.01 M betaine hydrochloride. Crystals appeared and grew to full size within a week at 21 °C.

Some crystals were looped and transferred to new drops of a solution containing 80% glycerol and 3 mM MTDB in addition to the original crystallization conditions to incorporate the PFSE ligand. Other crystals were grown in the presence of 10 mol equiv of ligand for each mole of RNA-Fab complex. In neither case was density for the ligand detected in the electron density map. Other crystals were looped and transferred to new drops containing the original crystallization conditions with added 20% glycerol (v/v) and 3 mM iridium hexammine as a cryo-protectant and to incorporate iridium hexammine into the crystal lattice. Iridium hexammine was synthesized in-house following the protocol established by Batey et al.^53^ A set of crystals were allowed to incubate for 24 h while another set of crystals were only allowed to incubate for 2 h before looping and freezing. The crystals incubated with iridium hexammine for only 2 h diffracted to higher resolution than those soaked for 24 h.

### Data Collection Processing and Analysis

The X-ray diffraction data sets were collected at the Advanced Photon Source NE-CAT section beamline 24-ID-C and 24-ID-E. Crystals soaked with iridium hexammine were shot with both the default wavelength of 0.979180 and a wavelength of 1.04040 to illicit anomalous diffraction of the iridium. All of the data sets were then integrated and scaled using its on-site RAPD automated programs (https://rapd.nec.aps.anl.gov). Initial SAD-MR phases were obtained from a data set which diffracted to only 3.27 Å using a partial molecular replacement (MR) solution of the Fab (PDB code: 6DB8) in Phenix Autosol.^38,40^ A low-resolution election density map was able to be calculated for a portion of the RNA from which a partial model of the FSE was built. This partial RNA model was used in addition to the fab model (PDB code: 6DB8) to find a molecular replacement solution for a higher resolution (2.09 Å) data set collected from a native unsoaked crystal. The electron density map using the MR phases of the partial RNA-Fab model were vastly improved and allowed for unambiguous model building nucleotide by nucleotide.^26,38^ The model was iteratively built and refined in COOT and Phenix Refine until the *R*_work_ and *R*_free_ could not be further improved.^39−41^ Water was automatically added during refinement and later validated visually in COOT^41^ according to the electron density map and difference map and potential hydrogen bonding interactions. All structure related figures were made in PyMOL^54^ (www.pymol.org), and figure labels were edited in Microsoft PowerPoint.

## Supporting Information Available

Four supplemental figures and one table that lists RNA constructs and DNA oligonucleotides used in this work (PDF)

## Accession Codes

Atomic coordinates and structure factors for the reported crystal structures have been deposited with the Protein Data Bank (PDB, https://www.rcsb.org/) under accession number 7MLX.

## References

[ref1] Warner K. D.; Hajdin C. E.; Weeks K. M. (2018) Principles for Targeting RNA with Drug-like Small *Molecules*. Nat. Rev. Drug Discovery 17 (8), 547–558. 10.1038/nrd.2018.93. 29977051 PMC6420209

[ref2] Shao Y.; Zhang Q. C. (2020) Targeting RNA Structures in Diseases with Small Molecules. Essays Biochem. 64 (6), 955–966. 10.1042/EBC20200011. 33078198 PMC7724634

[ref3] Park H. J.; Park S. J. (2012) Virtual Screening for RNA-Interacting Small Molecules. Biophysical approaches to translational control of gene expression. 1, 235–252. 10.1007/978-1-4614-3991-2_12.

[ref4] Bhatt P. R., Scaiola A., Loughran G., Leibundgut M., Kratzel A., McMillan A., O’ Connor K. M., Bode J. W., Thiel V., Atkins J. F., and Ban N. (2020) Structural Basis of Ribosomal Frameshifting during Translation of the SARS-CoV-2 RNA Genome, bioRxiv, 10.1101/2020.10.26.355099. PMC8168617 34029205

[ref5] Ritchie D. B.; Soong J.; Sikkema W. K. A.; Woodside M. T. (2014) Anti-Frameshifting Ligand Reduces the Conformational Plasticity of the SARS Virus Pseudoknot. J. Am. Chem. Soc. 136 (6), 2196–2199. 10.1021/ja410344b. 24446874

[ref6] Kelly J. A.; Woodside M. T.; Dinman J. D. (2021) Programmed −1 Ribosomal Frameshifting in Coronaviruses: A Therapeutic Target. Virology 554, 75–82. 10.1016/j.virol.2020.12.010. 33387787 PMC7833279

[ref7] Neupane K.; Munshi S.; Zhao M.; Ritchie D. B.; Ileperuma S. M.; Woodside M. T. (2020) Anti-Frameshifting Ligand Active against SARS Coronavirus-2 Is Resistant to Natural Mutations of the Frameshift-Stimulatory Pseudoknot. J. Mol. Biol. 432 (21), 5843–5847. 10.1016/j.jmb.2020.09.006. 32920049 PMC7483078

[ref8] Park S.-J.; Kim Y.-G.; Park H.-J. (2011) Identification of RNA Pseudoknot-Binding Ligand That Inhibits the −1 Ribosomal Frameshifting of SARS-Coronavirus by Structure-Based Virtual Screening. J. Am. Chem. Soc. 133 (26), 10094–10100. 10.1021/ja1098325. 21591761

[ref9] Rangan R.; Watkins A. M.; Chacon J.; Kretsch R.; Kladwang W.; Zheludev I. N.; Townley J.; Rynge M.; Thain G.; Das R. (2021) De Novo 3D Models of SARS-CoV-2 RNA Elements from Consensus Experimental Secondary Structures. Nucleic Acids Res. 49 (6), 3092–3108. 10.1093/nar/gkab119. 33693814 PMC8034642

[ref10] Omar S. I.; Zhao M.; Sekar R. V.; Moghadam S. A.; Tuszynski J. A.; Woodside M. T. (2021) Modeling the Structure of the Frameshift-Stimulatory Pseudoknot in SARS-CoV-2 Reveals Multiple Possible Conformers. PLoS Comput. Biol. 17 (1), e1008603 10.1371/journal.pcbi.1008603. 33465066 PMC7845960

[ref11] Lan T. C. T., Allan M. F., Malsick L. E., Khandwala S., Nyeo S. S. Y., Sun Y., Guo J. U., Bathe M., Griffiths A., and Rouskin S. (2021) Insights into the Secondary Structural Ensembles of the Full SARS-CoV-2 RNA Genome in Infected Cells, bioRxiv, 10.1101/2020.06.29.178343. PMC8891300 35236847

[ref12] Plant E. P.; Pérez-Alvarado G. C.; Jacobs J. L.; Mukhopadhyay B.; Hennig M.; Dinman J. D. (2005) A Three-Stemmed MRNA Pseudoknot in the SARS Coronavirus Frameshift Signal. PLoS Biol. 3 (6), e172 10.1371/journal.pbio.0030172. 15884978 PMC1110908

[ref13] Plant E. P.; Rakauskaite R.; Taylor D. R.; Dinman J. D. (2010) Achieving a Golden Mean: Mechanisms by Which Coronaviruses Ensure Synthesis of the Correct Stoichiometric Ratios of Viral Proteins. J. Virol. 84 (9), 4330–4340. 10.1128/JVI.02480-09. 20164235 PMC2863758

[ref14] Plant E. P.; Sims A. C.; Baric R. S.; Dinman J. D.; Taylor D. R. (2013) Altering SARS Coronavirus Frameshift Efficiency Affects Genomic and Subgenomic RNA Production. Viruses 5 (1), 279–294. 10.3390/v5010279. 23334702 PMC3564121

[ref15] Masters P. S. (2006) The Molecular Biology of Coronaviruses. Adv. Virus Res. 66, 193–292. 10.1016/S0065-3527(06)66005-3. 16877062 PMC7112330

[ref16] Brierley I.; Dos Ramos F. J. (2006) Programmed ribosomal frameshifting in HIV-1 and the SARS–CoV. Virus Res. 119 (1), 29–42. 10.1016/j.virusres.2005.10.008. 16310880 PMC7114087

[ref17] Chang K.-C. (2012) Revealing −1 Programmed Ribosomal Frameshifting Mechanisms by Single-Molecule Techniques and Computational Methods. Comput. Math. Methods Med. 2012, 569870 10.1155/2012/569870. 22545064 PMC3321566

[ref18] Atkins J. F.; Loughran G.; Bhatt P. R.; Firth A. E.; Baranov P. V. (2016) Ribosomal Frameshifting and Transcriptional Slippage: From Genetic Steganography and Cryptography to Adventitious Use. Nucleic Acids Res. 44 (15), 7007–7078. 10.1093/nar/gkw530. 27436286 PMC5009743

[ref19] Sun Y., Abriola L., Surovtseva Y. V., Lindenbach B. D., and Guo J. U. (2020) Restriction of SARS-CoV-2 Replication by Targeting Programmed −1 ribosomal Frameshifting in Vitro, bioRvix, 10.1101/2020.10.21.349225. PMC8256030 34185680

[ref20] Lin Z.; Gilbert R. J. C.; Brierley I. (2012) Spacer-Length Dependence of Programmed −1 or −2 Ribosomal Frameshifting on a U6A Heptamer Supports a Role for Messenger RNA (mRNA) Tension in Frameshifting. Nucleic Acids Res. 40 (17), 8674–8689. 10.1093/nar/gks629. 22743270 PMC3458567

[ref21] Ishimaru D.; Plant E. P.; Sims A. C.; Yount B. L. J.; Roth B. M.; Eldho N. V.; Pérez-Alvarado G. C.; Armbruster D. W.; Baric R. S.; Dinman J. D.; Taylor D. R.; Hennig M. (2013) RNA Dimerization Plays a Role in Ribosomal Frameshifting of the SARS Coronavirus. Nucleic Acids Res. 41 (4), 2594–2608. 10.1093/nar/gks1361. 23275571 PMC3575852

[ref22] Wu B.; Zhang H.; Sun R.; Peng S.; Cooperman B. S.; Goldman Y. E.; Chen C. (2018) Translocation Kinetics and Structural Dynamics of Ribosomes Are Modulated by the Conformational Plasticity of Downstream Pseudoknots. Nucleic Acids Res. 46 (18), 9736–9748. 10.1093/nar/gky636. 30011005 PMC6182138

[ref23] Caliskan N.; Katunin V. I.; Belardinelli R.; Peske F.; Rodnina M. V. (2014) Programmed – 1 Frameshifting by Kinetic Partitioning during Impeded Translocation. Cell 157 (7), 1619–1631. 10.1016/j.cell.2014.04.041. 24949973 PMC7112342

[ref24] Caliskan N.; Peske F.; Rodnina M. V. (2015) Changed in translation: mRNA recoding by – 1 programmed ribosomal frameshifting. Trends Biochem. Sci. 40 (5), 265–274. 10.1016/j.tibs.2015.03.006. 25850333 PMC7126180

[ref25] Dinman J. D. (2012) Mechanisms and Implications of Programmed Translational Frameshifting. Wiley Interdiscip. Rev. RNA 3 (5), 661–673. 10.1002/wrna.1126. 22715123 PMC3419312

[ref26] McCoy A. J.; Grosse-Kunstleve R. W.; Adams P. D.; Winn M. D.; Storoni L. C.; Read R. J. (2007) Phaser Crystallographic Software. J. Appl. Crystallogr. 40 (4), 658–674. 10.1107/S0021889807021206. 19461840 PMC2483472

[ref27] Halma M. T. J.; Ritchie D. B.; Cappellano T. R.; Neupane K.; Woodside M. T. (2019) Complex Dynamics under Tension in a High-Efficiency Frameshift Stimulatory Structure. Proc. Natl. Acad. Sci. U. S. A. 116 (39), 19500–19505. 10.1073/pnas.1905258116. 31409714 PMC6765238

[ref28] Ritchie D. B.; Foster D. A. N.; Woodside M. T. (2012) Programmed – 1 Frameshifting Efficiency Correlates with RNA Pseudoknot Conformational Plasticity, Not Resistance to Mechanical Unfolding. Proc. Natl. Acad. Sci. U. S. A. 109, 16167 10.1073/pnas.1204114109. 22988073 PMC3479558

[ref29] Halma M. T. J.; Ritchie D. B.; Woodside M. T. (2021) Conformational Shannon Entropy of MRNA Structures from Force Spectroscopy Measurements Predicts the Efficiency of −1 Programmed Ribosomal Frameshift Stimulation. Phys. Rev. Lett. 126 (3), 38102 10.1103/PhysRevLett.126.038102. 33543960

[ref30] Wu A.; Peng Y.; Huang B.; Ding X.; Wang X.; Niu P.; Meng J.; Zhu Z.; Zhang Z.; Wang J.; Sheng J.; Quan L.; Xia Z.; Tan W.; Cheng G.; Jiang T. (2020) Genome Composition and Divergence of the Novel Coronavirus (2019-NCoV) Originating in China. Cell Host Microbe 27 (3), 325–328. 10.1016/j.chom.2020.02.001. 32035028 PMC7154514

[ref31] Wacker A.; Weigand J. E; Akabayov S. R; Altincekic N.; Bains J. K.; Banijamali E.; Binas O.; Castillo-Martinez J.; Cetiner E.; Ceylan B.; Chiu L.-Y.; Davila-Calderon J.; Dhamotharan K.; Duchardt-Ferner E.; Ferner J.; Frydman L.; Furtig B.; Gallego J.; Grun J T.; Hacker C.; Haddad C.; Hahnke M.; Hengesbach M.; Hiller F.; Hohmann K. F; Hymon D.; de Jesus V.; Jonker H.; Keller H.; Knezic B.; Landgraf T.; Lohr F.; Luo L.; Mertinkus K. R; Muhs C.; Novakovic M.; Oxenfarth A.; Palomino-Schatzlein M.; Petzold K.; Peter S. A; Pyper D. J; Qureshi N. S; Riad M.; Richter C.; Saxena K.; Schamber T.; Scherf T.; Schlagnitweit J.; Schlundt A.; Schnieders R.; Schwalbe H.; Simba-Lahuasi A.; Sreeramulu S.; Stirnal E.; Sudakov A.; Tants J.-N.; Tolbert B. S; Vogele J.; Weiß L.; Wirmer-Bartoschek J.; Wirtz Martin M. A; Wohnert J.; Zetzsche H. (2020) Secondary Structure Determination of Conserved SARS-CoV-2 RNA Elements by NMR Spectroscopy. Nucleic Acids Res. 48 (22), 12415–12435. 10.1093/nar/gkaa1013. 33167030 PMC7736788

[ref32] Schlick T., Zhu Q., Dey A., Jain S., Yan S., and Laederach A. (2021) To Knot and Not: Multiple Conformations of the SARS-CoV-2 Frameshifting RNA Element, bioRxiv, 10.1101/2021.03.31.437955. PMC8315264 34283611

[ref33] Kelly J. A.; Olson A. N.; Neupane K.; Munshi S.; San Emeterio J.; Pollack L.; Woodside M. T.; Dinman J. D. (2020) Structural and functional conservation of the programmed −1 ribosomal frameshift signal of SARS coronavirus 2 (SARS-CoV-2). J. Biol. Chem. 295 (31), 10741–10748. 10.1074/jbc.AC120.013449. 32571880 PMC7397099

[ref34] Manfredonia I.; Nithin C.; Ponce-Salvatierra A.; Ghosh P.; Wirecki T. K.; Marinus T.; Ogando N. S.; Snijder E. J.; van Hemert M. J.; Bujnicki J. M.; Incarnato D. (2020) Genome-Wide Mapping of SARS-CoV-2 RNA Structures Identifies Therapeutically-Relevant Elements. Nucleic Acids Res. 48 (22), 12436–12452. 10.1093/nar/gkaa1053. 33166999 PMC7736786

[ref35] Schlick T.; Zhu Q.; Jain S.; Yan S. (2021) Structure-altering mutations of the SARS-CoV-2 frameshifting RNA element. Biophys. J. 120 (6), 1040–1053. 10.1016/j.bpj.2020.10.012. 33096082 PMC7575535

[ref36] Koldobskaya Y.; Duguid E. M.; Shechner D. M.; Suslov N. B.; Ye J.; Sidhu S. S.; Bartel D. P.; Koide S.; Kossiakoff A. A.; Piccirilli J. A. (2011) Portable RNA Sequence Whose Recognition by a Synthetic Antibody Facilitates Structural Determination. Nat. Struct. Mol. Biol. 18 (1), 100–106. 10.1038/nsmb.1945. 21151117 PMC3058332

[ref37] Koirala D.; Lewicka A.; Koldobskaya Y.; Huang H.; Piccirilli J. A. (2020) Synthetic Antibody Binding to a Preorganized RNA Domain of Hepatitis C Virus Internal Ribosome Entry Site Inhibits Translation. ACS Chem. Biol. 15 (1), 205–216. 10.1021/acschembio.9b00785. 31765566 PMC7919738

[ref38] Liebschner D.; Afonine P. V.; Baker M. L.; Bunkóczi G.; Chen V. B.; Croll T. I.; Hintze B.; Hung L. W.; Jain S.; McCoy A. J.; Moriarty N. W.; Oeffner R. D.; Poon B. K.; Prisant M. G.; Read R. J.; Richardson J. S.; Richardson D. C.; Sammito M. D.; Sobolev O. V.; Stockwell D. H.; Terwilliger T. C.; Urzhumtsev A. G.; Videau L. L.; Williams C. J.; Adams P. D. (2019) Macromolecular Structure Determination Using X-Rays, Neutrons and Electrons: Recent Developments in Phenix. Acta Crystallogr. Sect. D 75 (10), 861–877. 10.1107/S2059798319011471. PMC6778852 31588918

[ref39] Williams C. J.; Headd J. J.; Moriarty N. W.; Prisant M. G.; Videau L. L.; Deis L. N.; Verma V.; Keedy D. A.; Hintze B. J.; Chen V. B.; Jain S.; Lewis S. M.; Arendall W. B. 3rd; Snoeyink J.; Adams P. D.; Lovell S. C.; Richardson J. S.; Richardson D. C. (2018) MolProbity: More and Better Reference Data for Improved All-Atom Structure Validation. Protein Sci. 27 (1), 293–315. 10.1002/pro.3330. 29067766 PMC5734394

[ref40] Afonine P. V.; Grosse-Kunstleve R. W.; Echols N.; Headd J. J.; Moriarty N. W.; Mustyakimov M.; Terwilliger T. C.; Urzhumtsev A.; Zwart P. H.; Adams P. D. (2012) Towards Automated Crystallographic Structure Refinement with Phenix.Refine. Acta Crystallogr. Acta Crystallogr., Sect. D: Biol. Crystallogr. 68 (4), 352–367. 10.1107/S0907444912001308. 22505256 PMC3322595

[ref41] Emsley P.; Lohkamp B.; Scott W. G.; Cowtan K. (2010) Features and Development of Coot. Acta Crystallogr., Sect. D: Biol. Crystallogr. 66 (4), 486–501. 10.1107/S0907444910007493. 20383002 PMC2852313

[ref42] Gholamalipour Y.; Karunanayake Mudiyanselage A.; Martin C. T (2018) 3′ End Additions by T7 RNA Polymerase are RNA Self-Templated, Distributive and Diverse in Character-RNA-Seq Analyses. Nucleic Acids Res. 46 (18), 9253–9263. 10.1093/nar/gky796. 30219859 PMC6182178

[ref43] Zhang K., Zheludev I. N., Hagey R. J., Wu M. T.-P., Haslecker R., Hou Y. J., Kretsch R., Pintilie G. D., Rangan R., Kladwang W., Li S., Pham E. A., Bernardin-Souibgui C., Baric R. S., Sheahan T. P., D Souza V., Glenn J. S., Chiu W., and Das R. (2020) Cryo-Electron Microscopy and Exploratory Antisense Targeting of the 28-KDa Frameshift Stimulation Element from the SARS-CoV-2 RNA Genome, bioRxiv, 10.1101/2020.07.18.209270. PMC8848339 34426697

[ref44] Chen H.; Meisburger S. P.; Pabit S. A.; Sutton J. L.; Webb W. W.; Pollack L. (2012) Ionic Strength-Dependent Persistence Lengths of Single-Stranded RNA and DNA. Proc. Natl. Acad. Sci. U. S. A. 109 (3), 799–804. 10.1073/pnas.1119057109. 22203973 PMC3271905

[ref45] Iserman C., Roden C., Boerneke M., Sealfon R., McLaughlin G., Jungreis I., Park C., Boppana A., Fritch E., Hou Y. J., Theesfeld C., Troyanskaya O. G., Baric R. S., Sheahan T. P., Weeks K., and Gladfelter A. S. (2020) Specific Viral RNA Drives the SARS CoV-2 Nucleocapsid to Phase Separate, bioRxiv, 10.1101/2020.06.11.147199.

[ref46] Harrington H. R.; Zimmer M. H.; Chamness L. M.; Nash V.; Penn W. D.; Miller T. F.; Mukhopadhyay S.; Schlebach J. P. (2020) Co-translational folding stimulates programmed ribosomal frameshifting in the alphavirus structural polyprotein. J. Biol. Chem. 295 (20), 6798–6808. 10.1074/jbc.RA120.012706. 32169904 PMC7242702

[ref47] Huston N. C.; Wan H.; Strine M. S.; de Cesaris Araujo Tavares R.; Wilen C. B.; Pyle A. M. (2021) Comprehensive in vivo secondary structure of the SARS-CoV-2 genome reveals novel regulatory motifs and mechanisms. Mol. Cell 81, 584–598. 10.1016/j.molcel.2020.12.041. 33444546 PMC7775661

[ref48] Chen G.; Chang K.-Y.; Chou M.-Y.; Bustamante C.; Tinoco I. (2009) Triplex Structures in an RNA Pseudoknot Enhance Mechanical Stability and Increase Efficiency of – 1 Ribosomal Frameshifting. Proc. Natl. Acad. Sci. U. S. A. 106 (31), 12706–12711. 10.1073/pnas.0905046106. 19628688 PMC2722267

[ref49] Giedroc D. P.; Cornish P. V. (2009) Frameshifting RNA Pseudoknots: Structure and Mechanism. Virus Res. 139 (2), 193–208. 10.1016/j.virusres.2008.06.008. 18621088 PMC2670756

[ref50] Neupane K. P.; Zhao M.; Hoffer N. Q.; Lyons A.; Munshi S.; Ritchie D.; Woodside M. T. (2021) Structural Dynamics of SARS-CoV-2 Frameshift Signal Studied by Single-Molecule Force Spectroscopy Reveal Topologically Distinct Conformers. Biophys. J. 120 (3), 314a–314a. 10.1016/j.bpj.2020.11.1987. PMC8346527 34362921

[ref51] Xu X.; Zhao P.; Chen S.-J. (2014) Vfold: A Web Server for RNA Structure and Folding Thermodynamics Prediction. PLoS One 9 (9), e107504 10.1371/journal.pone.0107504. 25215508 PMC4162592

[ref52] Rio D. C. (2013) Expression and Purification of Active Recombinant T7 RNA Polymerase from E. Coli. Cold Spring Harb. Protoc. 2013 (11), pdb.prot078527 10.1101/pdb.prot078527. 24184761

[ref53] Batey R. T.; Kieft J. S. (2016) Soaking Hexammine Cations into RNA Crystals to Obtain Derivatives for Phasing Diffraction Data. Methods Mol. Biol. 1320, 219–232. 10.1007/978-1-4939-2763-0_14. 26227046 PMC4586159

[ref54] The PyMOL Molecular Graphics System, version 2.0, Schrödinger, LLC.

[ref55] Nilsen T. W. (2014) 3′-End Labeling of RNA with [5′- ^32^ P]Cytidine 3′,5′-Bis(Phosphate) and T4 RNA Ligase 1. Cold Spring Harb. Protoc. 2014 (4), 444–446. 10.1101/pdb.prot080713. 24692494

[ref56] Homemade [5′-^32^P]pCp. (2014) Cold Spring Harb. Protoc. 2014, pdb.rec082123, 10.1101/pdb.rec082123.

[ref57] Grosjean H.; Keith G.; Droogmans L. (2004) Detection and quantification of modified nucleotides in RNA using thin-layer chromatography. Methods Mol. Biol. 265, 357–91. 10.1385/1-59259-775-0:357. 15103084

[ref58] Cao C.; Cai Z.; Xiao X.; et al. (2021) The architecture of the SARS-CoV-2 RNA genome inside virion. Nat. Commun. 12, 3917 10.1038/s41467-021-22785-x. 34168138 PMC8225788

[ref59] Wu Q.; Zhang Y.; Lü H.; Wang J.; He X.; Liu Y.; Ye C.; Lin W.; Hu J.; Ji J.; Xu J.; Ye J.; Hu Y.; Chen W.; Li S.; Wang J.; Wang J.; Bi S.; Yang H. (2003) The E protein is a multifunctional membrane protein of SARS-CoV. Genomics, Proteomics Bioinf. 1 (2), 131–44. 10.1016/S1672-0229(03)01017-9. PMC5172412 15626343

