# Supplementary material for: The SARS-CoV-2 Programmed −1 Ribosomal Frameshifting Element Crystal Structure Solved to 2.09 Å Using Chaperone-Assisted RNA Crystallography
Source: ACS Chem Biol. 2021 Jul 30;16(8):1469–81. doi: 10.1021/acschembio.1c00324 (PMC8353986; doi:10.1021/acschembio.1c00324)
Supplement: Supplementary file 1 — cb1c00324_si_001.pdf [file cb1c00324_si_001.pdf]

Supplementary Information

The SARS-CoV-2 Programmed -1 Ribosomal Frameshifting Element Crystal Structure Solved to 2.09 Å Using Chaperone-Assisted RNA Crystallography

Christina Roman [1] #, Anna Lewicka [1] #, Deepak Koirala [3], Nan-Sheng Li [1], Joseph A. Piccirilli* [1,2]

[1] Department of Biochemistry and Molecular Biology, The University of Chicago, Chicago, Illinois 60637, USA

[2] Department of Chemistry, The University of Chicago, Chicago, Illinois 60637, USA.

[3] Department of Chemistry and Biochemistry, University of Maryland Baltimore County (UMBC), Baltimore, Maryland 21250, USA

* Corresponding author email: jpicciri@uchicago.edu

# These authors contributed equally to this work and both should be considered first authors

Supplementary Figures

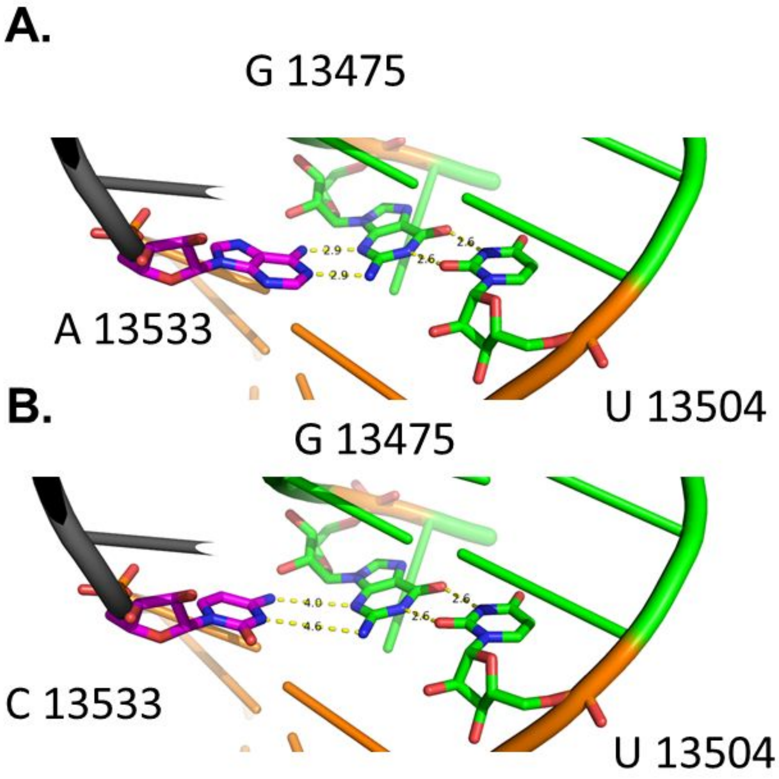

Supplementary Fig. 1 Comparison of position 13533 as an A and as a C. (A) Close up view of the SARS-CoV-2 PFSE showing position A13533, modeled as magenta-colored sticks, forming hydrogen bonds with G13475, which is paired with U13504 (both green). (B) The image displayed in A but with position 13533 mutated to C to show the potential to form the same hydrogen bonding interactions as (A).

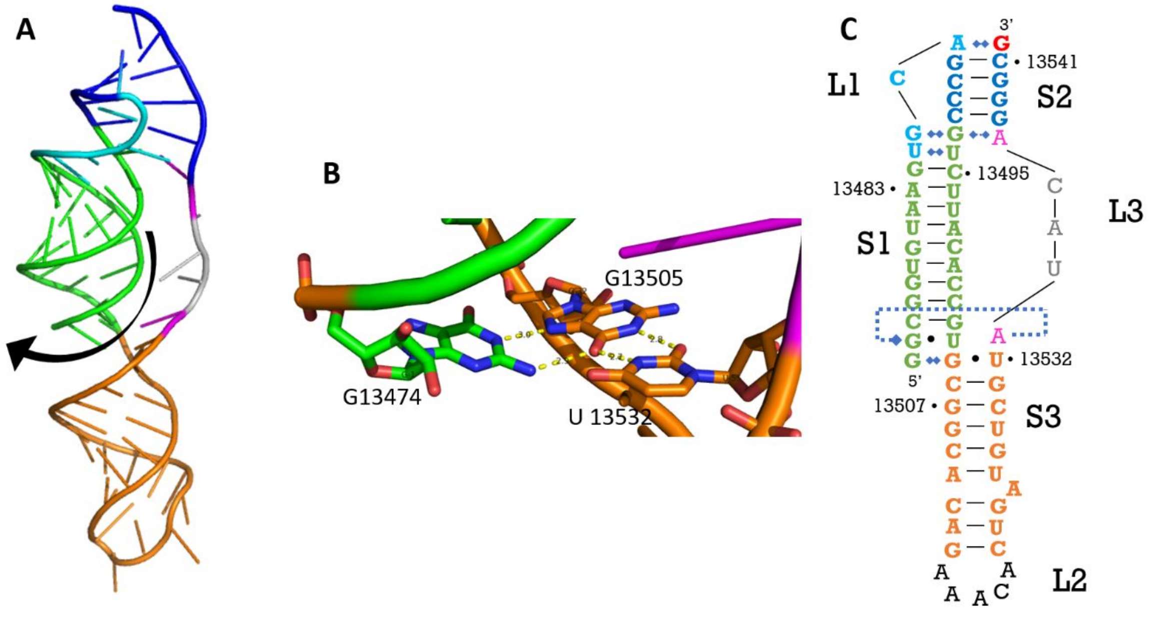

Supplementary Fig. 2 Observed 5' end interactions of the PFSE. (A) 5' end threading of the PFSE illustrated by an arrow. (B) Hydrogen bonding interactions of G13474 with G13505-U13532 wobble pair. (C) Location of panel (B) in relation to the secondary structure map.

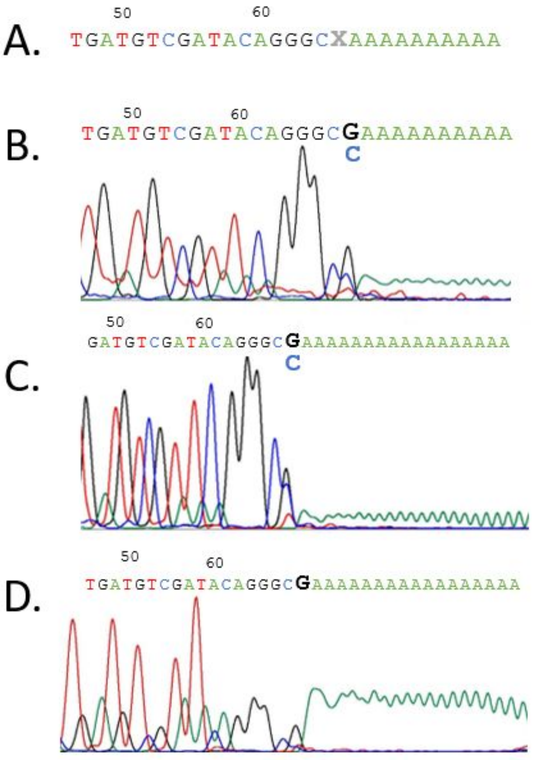

Supplementary Fig. 3 Chromatograms of PFSE RNA sequencing. (A) Sequence of the PFSE BL3-6 RNA with unknown nucleotide in position 65 flanked by poly(A) tail. (B) PFSE BL3-6 RNA sequenced after transcription. (C) PFSE BL3-6 RNA sequenced from the crystallization drop mother liquor. (D) PFSE BL3-6 RNA sequenced from the crystal.

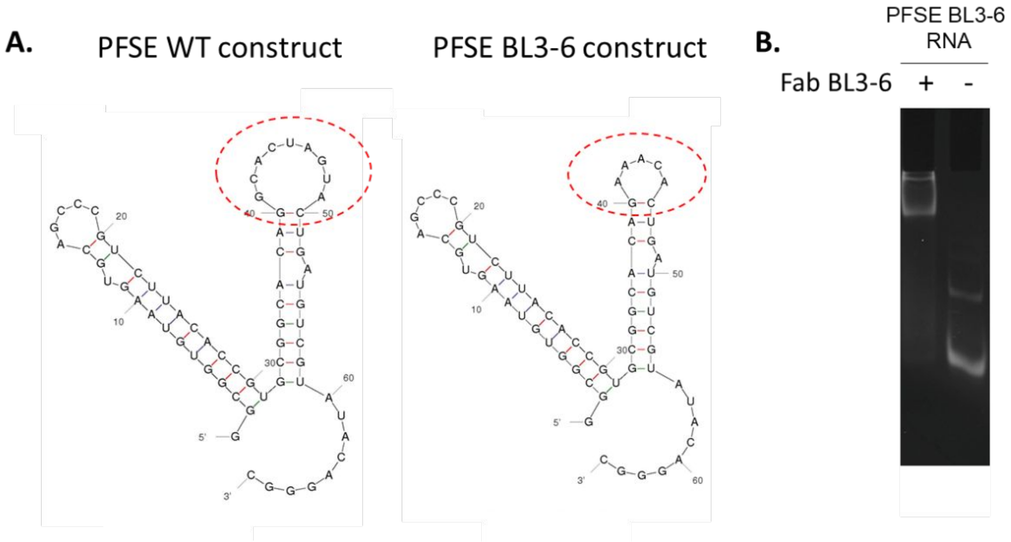

Supplementary Fig. 4 Construct validation. (A) V-fold[2] predictions of the wild type PFSE sequence and the PFSE BL3-6 sequence demonstrating that mutating Loop 2 to AAACA would not be expected to create new long distance base pairing interactions that could interfere with the native structure. (B) EMSA in 10% native gel demonstrating RNA shift in the presence of Fab BL3-6.

Sequences of RNA Constructs and DNA Oligonucleotides

Construct Name	Sequence
WT PFSE RNA	5’ GGC GGU GUA AGU GCA GCC CGU CUU ACA CCG UGC GGC ACA GGC ACU AGU ACU GAU GUC GUA UAC AGG GC 3’
BL3-6 PFSE	5’ GGC GGU GUA AGU GCA GCC CGU CUU ACA CCG UGC GGC ACA G AAACA CU GAU GUC GUA UAC AGG GC 3’
WT PFSE transcription template DNA oligo	5’ GCG TAA TAC GAC TCA CTA TA GGC GGT GTA AGT GCA GCC CGT CTT ACA CCG TGC GGC ACA GGC ACT AGT ACT GAT GTC GTA TAC AGG GC 3’
WT PFSE transcription template DNA forward primer	5’ GCGTAATACGACTCACTATAGG 3’
WT PFSE transcription template DNA reverse primer	5’ (2’O-Me)-GCCCTGTATACGACATCAG 3’
BL3-6 PFSE transcription template DNA oligo	5’ GCG TAA TAC GAC TCA CTA TA GGC GGT GTA AGT GCA GCC CGT CTT ACA CCG TGC GGC ACA G AAACA CT GAT GTC GTA TAC AGG GC 3’
BL3-6 PFSE transcription template DNA forward primer	5’ GCGTAATACGACTCACTATAGG 3’
BL3-6 PFSE transcription template DNA reverse primer	5’ (2’O-Me)-GCCCTGTATACGACATCAG 3’
Reverse transcription reverse primer (M13 for + 18 T’s)	5’ GTAAAACGACGGCCAGTTTTTTTTTTTTTTTTTT 3’
Reverse transcription forward primer (M13)	5’ GTAAAACGACGGCCAGT 3’
Reverse primer (PFSE specific)	5’ GGCGGTGTAAGTGCAG 3’

Supplementary References

(1) Xu, X.; Zhao, P.; Chen, S.-J. Vfold: A Web Server for RNA Structure and Folding Thermodynamics Prediction. *PLoS One* **2014**, 9 (9), e107504. https://doi.org/10.1371/journal.pone.0107504.
